# TCGA based integrated genomic analyses of ceRNA network and novel subtypes revealing potential biomarkers for the prognosis and target therapy of tongue squamous cell carcinoma

**DOI:** 10.1371/journal.pone.0216834

**Published:** 2019-05-29

**Authors:** Zaiye Li, Canhua Jiang, Yongxiang Yuan

**Affiliations:** Department of Oral and Maxillofacial Surgery, Xiangya Hospital, Central South University, Changsha, Hunan, China; Queen Mary University of London, UNITED KINGDOM

## Abstract

**Objectives:**

The study aimed to investigate the ceRNA network in biological development of Tongue Squamous Cell Carcinoma (TSCC) and to identify novel molecular subtypes of TSCC to screen potential biomarkers for target therapy and prognosis by using integrated genomic analysis based on The Cancer Genome Atlas (TCGA) database.

**Material and methods:**

Data on gene expressions were downloaded from TCGA and GEO database. Differentially expressed RNAs(DERNAs) were shown by DESeq2 package in R. Functional enrichment analysis of DEmRNAs was performed using clusterprofilers in R. PPI network was established by referring to String website. Survival analysis of DERNAs was carried out by survival package in R. Interactions among mRNAs, miRNAs and lncRNAs were obtained from Starbase v3.0 and used to construct ceRNA network. Consensus Cluster Plus package was applied to identify molecular subtypes. All key genes were validated by comparing them with GEO microarray data. Statistical analyses of clinical features among different subtypes were performed using SPSS 22.0.

**Results:**

A total of 2907 mRNAs (1366 up-regulated and 1541 down-regulated), 191miRNAs (98 up-regulated and 93 down-regulated) and 1831 lncRNAs (1151 up-regulated and 680 down-regulated) were identified from tumor and normal tissues. A ceRNA network was successfully constructed and 15 DEmRNAs, 1 DEmiRNA, 2 DElncRNAs associated with prognosis were employed. Furthermore, we firstly identified 2 molecular subtypes, basal and differentiated, and found that differentiated subtype consumed less alcohol and was related to a better overall survival.

**Conclusion:**

The study constructed a ceRNA network and identified molecular subtypes of TSCC, and our findings provided a novel insight into this intractable cancer and potential therapeutic targets and prognostic indicators.

## Introduction

Oral squamous cell carcinoma (OSCC), among others, is one of the most frequent cancers in the world. The major type of OSCC is tongue squamous cell carcinoma, the incidence which accounts for 25%~40% [1]. OSCC is characterized by a rapid local invasion, an early lymph node metastasis and a poor prognosis [2]. According to a retrospective analysis by Nair [3], TSCC was clinically different from other oral cancers due to its pathological factors, suggesting that TSCC could be regarded as a particular type of OSCC. The occurrence of TSCC could be attributed to tobacco, alcohol and areca nut [4]. Although great efforts have been made in the advances in surgical techniques, chemo-radiotherapy and some other target therapies, the overall survival of TSCC still hovers around 50% [5]. Thus, it is crucial to reveal the underlying biological mechanism and different molecular subtypes of TSCC associated with prognosis in order to discover novel biomarkers for target therapy and prognosis prediction.

MRNAs and some non-coding RNAs, including microRNAs(miRNAs) and long non-coding RNAs (lncRNAs), play an important role in the initiation and progression of TSCC. MiRNA, which is a family of functional noncoding RNA molecules with a length of 20–25 nucleotides, widely participate in post-transcriptional regulation by targeting mRNAs. A series of miRNAs have been demonstrated as oncogenes or tumor suppressors of TSCC [6,7]. LncRNAs, which are defined as endogenous non-coding RNAs that are longer than 200 nucleotides, regulate biological processes including transcriptional regulation, cell metabolism and RNA modification in tumorigenesis and metastasis of TSCC [8,9]. On the basis of the function of miRNAs and lncRNAs, Salmena et al put forward a hypothesis of competing endogenous RNA (ceRNA) across transcriptome, in which all types of RNA transcripts were believed to be able to act as a ceRNA through microRNA response elements (MREs) that are accessible to microRNA binding [10]. According to such a hypothesis, a great number of studies further demonstrated the importance of ceRNA in the occurrence and development of different cancers. For example, Zhou et al used GEO database to construct a miRNA-mRNA network of TSCC [11]. Nohata et al identified a prognostic lncRNAs malignant progression of TSCC [12]. The ceRNA network of OSCC was first established by Simin Li in 2017[13]. However, to the best of our knowledge, no study has been conducted on ceRNA network of TSCC.

In addition to the regulation of ceRNA network in carcinogenesis, different clinical or molecular subtypes have been increasingly shown to be associated with different clinical and pathological factors. Walter et al identified 4 gene expression subtypes of head and neck squamous cell carcinoma (HNSCC) [14], and to further expand Walter’s study, Zevallos [15] et al carried out gene expression analysis and demonstrated 4 subtypes to predict nodal metastasis and survival in human papilloma virus (HPV)–negative OSCC. However, we still lack a classification of TSCC subtypes.

Therefore, the aim of present study is to investigate the hidden crosstalk among various RNAs in TSCC via ceRNA network construction and to identify molecular subtypes of TSCC that can be used to predict prognosis. Furthermore, we identified that several genes with differential expressions from tumor and normal tissues were significantly associated with overall survival by integrated analysis. Our findings provided a novel insight into the biological process of TSCC and potential biomarkers for target therapy and survival prediction.

## Methods and materials

### 1. Data collection and preprocessing

Gene expression data (RNA sequencing profiles and miRNA profiling) and corresponding clinical data of TSCC were obtained from TCGA database (https://portal.gdc.cancer.gov/), and 126 TSCC samples and 13 normal controlled samples were collected. Among these data, data on mRNA and lncRNA expressions were obtained from Illumina HiSeqRNASeq platforms, while miRNA data were collected from Illumina HiSeqmiRNASeq platforms.

RNA sequencing profiles based on TCGA were preprocessed by pre-filtered low count genes (total counts<10). MRNAs and lncRNAs were encoded according to GENCODE Release 29 (GRCh38.p12) (https://www.gencodegenes.org/human/). miRNAs were annotated based on miRbase v22 (http://www.mirbase.org/index.shtml#opennewwindow).

3 gene expression profiles of TSCC (GSE30784, GSE13601 and GSE28100) were downloaded from Gene Expression Omnibus (GEO) database (http://www.ncbi.nlm.nih.gov/geo/) by searching the term “tongue squamous cell carcinoma” (January 2019). GSE30784 and GSE13601 were determined based on Affymetrix Human Genome U133 Plus 2.0 Array and U95 Version 2 Array. The platform of GSE28100 was Agilent-021827 Human miRNA Microarray (V3) (miRBase release 12.0 miRNA ID version).

### 2. Identification of differentially expressed mRNAs, miRNAs, and lncRNAs in TSCC

The differentially expressed lncRNA (DElncRNA), mRNA (DEmRNA), miRNA (DEmiRNA) in TSCC samples and normal controlled samples were identified using DESeq2 package of R software (Version 3.8; http://www.bioconductor.org/packages/release/bioc/html/DESeq2.html). P-value was adjusted to false discovery rate (FDR). |log2 fold change (FC) | >1.5 and P-value<0.05 were set as the cutoff criteria. The heatmaps were plotted based on pheatmap package of R.

### 3. Functional enrichment analysis of GO annotation and KEGG pathways

ClusterProfiler v3.8 package of R was used to analyze and visualize functional profiles (Gene Ontology (GO) annotation and Kyoto Encyclopedia of Genes and Genomes (KEGG) pathway) of the genes in order to determine shared functions among DEmRNAs. P<0.05 was considered as a threshold of GO and KEGG enrichment analysis.

### 4. Establishment of protein–protein interaction (PPI) network

To understand the underlying interaction of DEmRNAs, the STRING website was employed to construct the PPI network, which was visualized by the Cytoscape software v3.6.1.

### 5. DEmRNAs, DElncRNAs, and DEmiRNAs associated with prognosis

Survival analysis was carried out by using the survival package of R to help assess the prognostic value of differentially expressed RNAs in TSCC patients. All samples were divided into either the high-expression group(>median) or the low-expression group(<median) in terms of the expression of each DEmRNA, DElncRNA, and DEmiRNA. The Survival curves were plotted using the Kaplan-Meier method. The log-rank test was adopted to assess statistical significance. P<0.05 was considered as statistically significant.

### 6. Prediction of lncRNA–miRNA and miRNA–mRNA interactions

We predict the interaction between DElncRNA and DEmiRNA or DEmRNA and DEmiRNA by using starBase v3.0, which determined more than 1.1 million miRNA-ncRNA, 2.5 million miRNA-mRNA and 1.5 million RNA-RNA interactions from multi-dimensional sequencing data. In addition, the prediction results of miRanda, Targerscan and miRmap were integrated by starBase. Only the regulatory pairs of DEmiRNAs and DEGs, DElncRNAs and DEmiRNAs had opposite expressions and were therefore included in the present study.

### 7. Construction of ceRNAs regulatory network

According to ceRNA theory, the selected interaction of DEmiRNAs and DEmRNAs and of DElncRNAs and DEmiRNAs were integrated to construct the DElncRNAs-DEmiRNAs-DEmRNAs ceRNA network using Cytoscape software v3.6.1.

### 8. Validation of expressions of crucial DEmRNAs, DElncRNAs, and DEmiRNAs basing on ceRNA network and prognosis analysis

GEO2R (http://www.ncbi.nlm.nih.gov/geo/geo2r/) is an interactive web analysis tool for GEO data to identify genes with differential expressions among different groups provided by GEO database. We applied GEO2R to confirm key RNAs. Crucial DEmRNAs and DElncRNAs were verified in GSE30784 and GSE13601 and GSE28100 was used to validate crucial DEmiRNAs.

### 9. Copy number variations (CNV), somatic mutation (SNV) and methylation analysis of hub genes

To determine the possible regulatory mechanism of crucial RNAs with differential expressions from TSCC and normal tissue, CNV and methylation data analysis on selected RNAs were performed by cor package of R software. Pearson correlation analysis was carried out to identify whether they had correlated relationship with Pearson correlation coefficient >0.40, which was set as a cutoff. SNV analysis was performed to excavate whether there existed some mutations of genes that possibly regulated genes, which were most significantly related to prognosis, by using Fisher test. P value < 0.05 was considered as statistically significant.

### 10. Molecular subtypes of TSCC classification

To find out if some molecular subtypes of TSCC were associated with prognosis, we classify TSCC subtypes in terms of gene expression from TCGA using ConsensusClusterPlus package of R. The top 2000 variable genes were reduced for integrative clustering analysis by median absolute deviation. All sample were divided into k (2 to 6) groups for determining a stable classification by k value analysis.

### 11. Clinical features description and survival analysis of different subtypes

Clinical information (i.e. clinical stage, T stage, N stage, smoking history, alcohol history, nodal extracapsular spread) was collected. Chi-square test was used to assess statistical significance between each subtype with p<0.05, which was set as the cut-off criterion. Survival analyses of patients with different subtypes were performed subsequently using a log-rank test by survival package of R. P value <0.05 was set as a threshold.

### 12. Identification of differential expression of mRNAs, miRNAs, lncRNAs between different subtypes of TSCC associated with prognostic DERNAs and functional enrichment analysis of GO and KEGG

To understand the intrinsic biological features between different molecular subtypes, we identified mRNAs, lncRNAs and miRNAs with differential expressions. Among these DERNAs, genes overlapped with prognostic genes mentioned above were considered as potential prognostic predictors for TSCC, according to different subtypes. Functional enrichment analyses were carried out to excavate biological mechanism and to define gene expression subtypes by clusterprofiler mentioned above. P<0.05 was considered as a cutoff of GO and KEGG enrichment analysis.

## Results

### 1. DElncRNAs, DEmiRNAs, and DEmRNAs in TSCC

**Fig 1:**
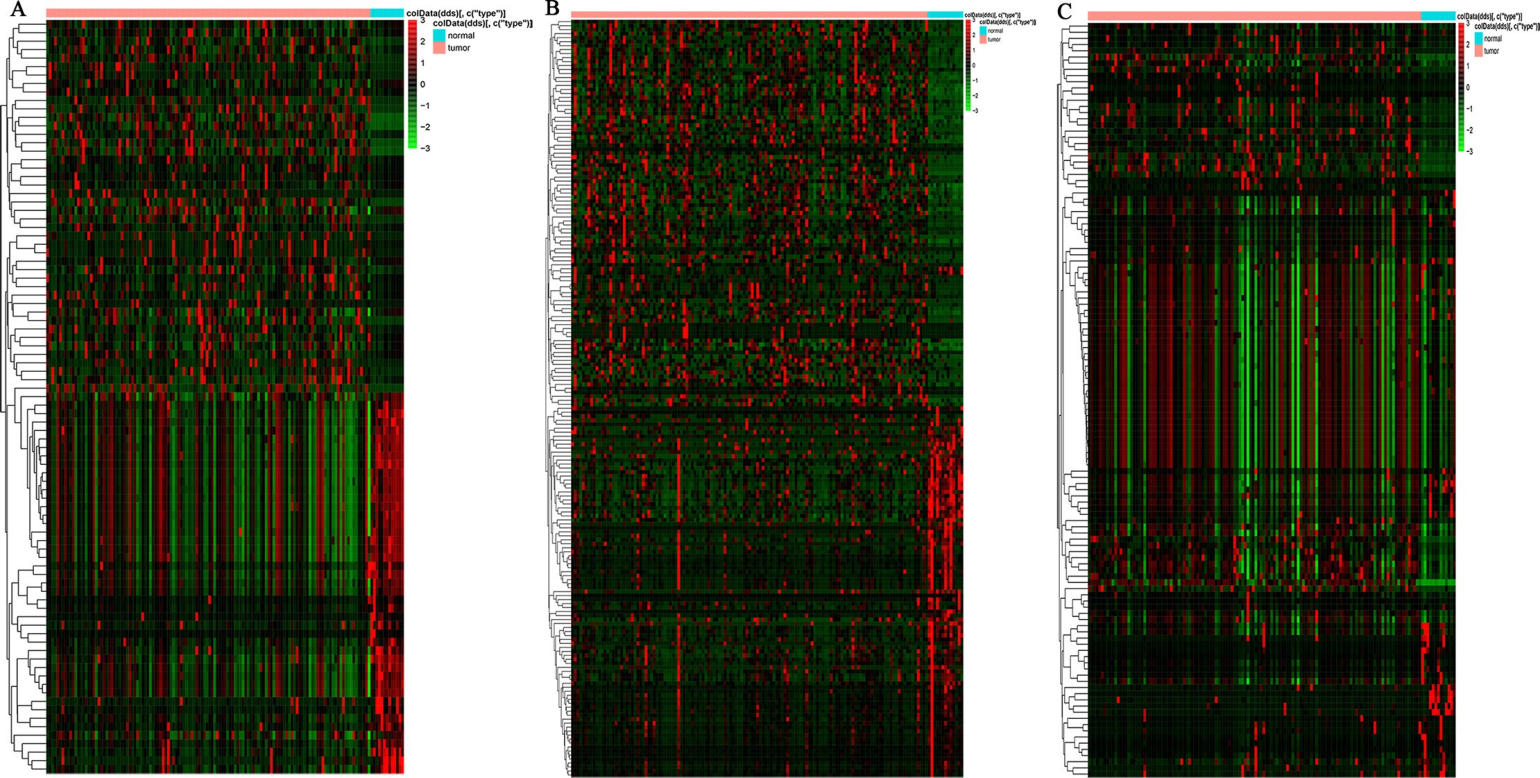
Heatmap of top 100 variable of differentially expressed RNAs between TSCC and non-tumor tissues. (A) DElncRNAs, (B) DEmiRNAs, (C) DEmRNAs.

A total of 2907 mRNAs (1366 up-regulated and 1541 down-regulated), 191miRNAs (98 up-regulated and 93 down-regulated) and 1831 lncRNAs (1151 up-regulated and 680 down-regulated) were identified in mRNA-seq and miRNAseq data between |log2 fold change (FC) |>1.5 and P value < 0.05. RNAs with differential expressions were visualized in heatmap (Fig 1). Top 10 DElncRNAs, DEmiRNAs and DEmRNAs were list in Table 1.

**Table 1:** Top 10 DElncRNAs, DEmiRNAs and DEmRNAs between tumor and normal tissues in TSCC.

	Symbol	Log2 Fold Change	P value	Type
lncRNA	LINC01322	8.484446	2.6E-24	Up
	G2E3-AS1	7.404014	1.99E-10	Up
	LINC01614	7.147533	8.21E-19	Up
	BX322234.2	6.685120	3.36E-13	Up
	LINC02434	6.651148	7.58E-12	Up
	AC011632.1	6.614810	4.80E-09	Up
	LINC02577	6.504801	2.32E-34	Up
	AFAP1-AS1	6.460035	1.49E-17	Up
	DLGAP1-AS5	-6.304910	1.42E-10	Down
	LINC02582	6.284453	1.03E-07	Up
miRNA	miR-615-3p	5.633801	7.38E-17	Up
	miR-135a-5p	-5.447620	1.57E-14	Down
	miR-105-5p	5.239907	6.10E-08	Up
	miR-1269b	5.104939	0.001494	Up
	miR-1251-5p	-5.07307	1.46E-05	Down
	miR-4652-5p	4.884050	6.15E-10	Up
	miR-767-5p	4.811304	7.29E-07	Up
	miR-1269a	4.717552	5.45E-07	Up
	miR-375-3p	-4.59202	1.88E-13	Down
	miR-196a-5p	4.518151	1.05E-20	Up
	miR-1910-5p	4.302478	2.11E-10	Up
mRNA	SMR3B	-13.322939	3.39E-16	Down
	PRH2	-12.899204	8.68E-45	Down
	BPIFA2	-12.664771	4.21E-18	Down
	PIP	-12.656535	3.43E-19	Down
	C6orf58	-11.223360	1.12E-84	Down
	ZG16B	-10.726593	4.64E-49	Down
	KRT36	-10.329547	1.64E-39	Down
	STATH	-9.9115524	4.04E-18	Down
	PRR4	-9.6244041	3.05E-50	Down
	MAGEB2	8.4345422	1.57E-09	Up

### 2. Functional enrichment analysis of gene ontology and KEGG pathways

**Fig 2:**
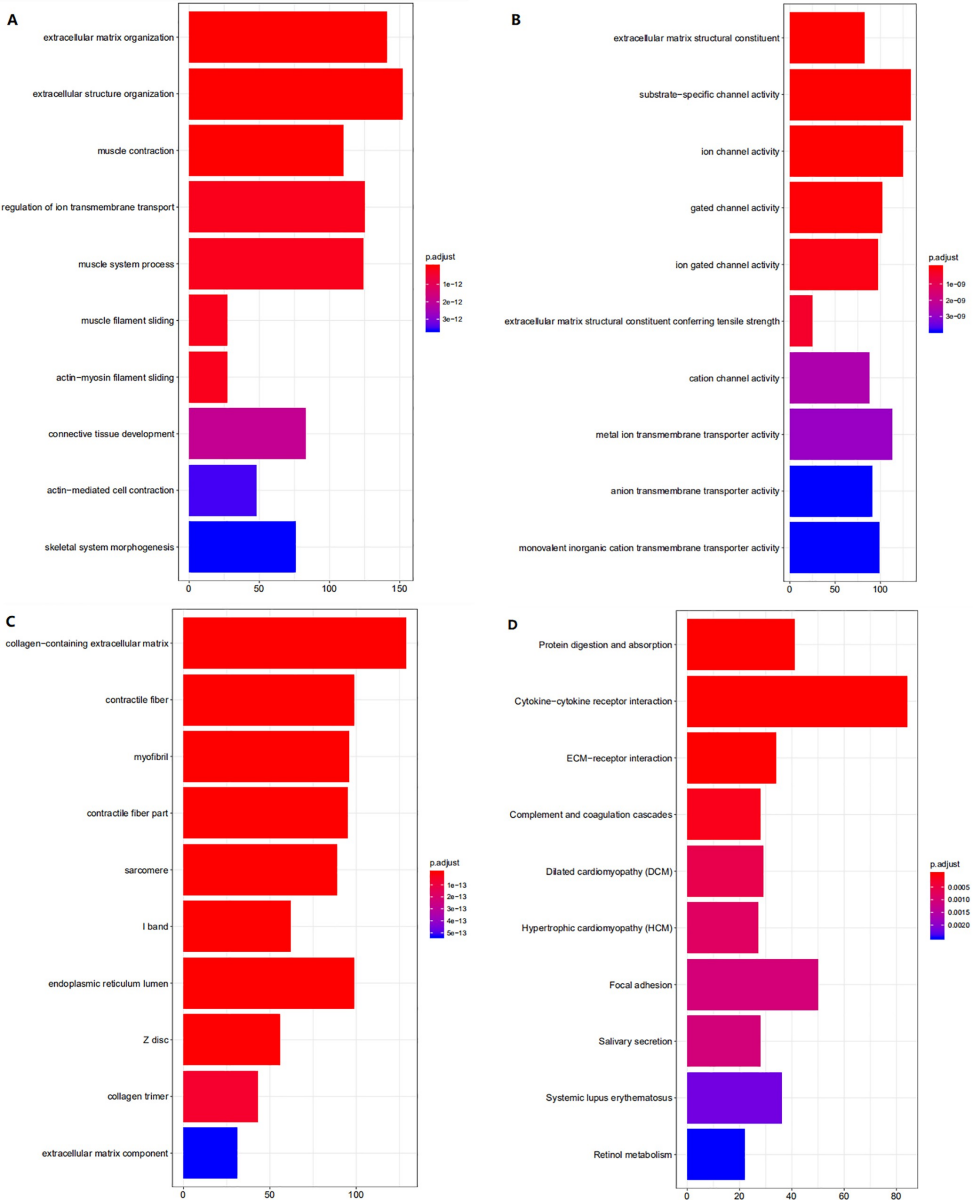
The top 10 enrichment scores in KEGG pathway and GO enrichment analysis of the DEmRNA. (A)Biological process of DEmRNAs; (B) Molecular function of DEmRNAs; (C) cellular component of DEmRNAs; (D) KEGG pathway of DEmRNAs.

2907 DEmRNAs were involved in the functional enrichment analysis using clusterProfiler package of R. GO annotation consists of biological process (BP), cellular component (CC), molecular function (MF). The top 50 GO analyses results were list in S1 Table. These results (Fig 2) revealed that DEmRNAs were enriched in 1313 BPs, most of which took part in extracellular organization, ion transmembrane transport, muscle biological process and epidermal cell differentiation. MF analysis indicated that these DEmRNAs were significantly associated with 257 MFs, including channel activity, DNA-binding transcription activator activity, RNA polymerase II-specific, actin binding, enzyme inhibitor activity and cytokine activity. We found that the most enriched CC was collagen-containing extracellular matrix. KEGG pathway analysis (Fig 2 and S2 Table) showed that a total of 50 pathways were highly enriched, which was consistent to DEmRNAs. These pathways were mainly involved in Cytokine-cytokine receptor interaction, PI3K-Akt signaling pathway, Human papillomavirus infection, Focal adhesion and Calcium signaling pathway.

### 3. Protein-protein network analysis

**Fig 3:**
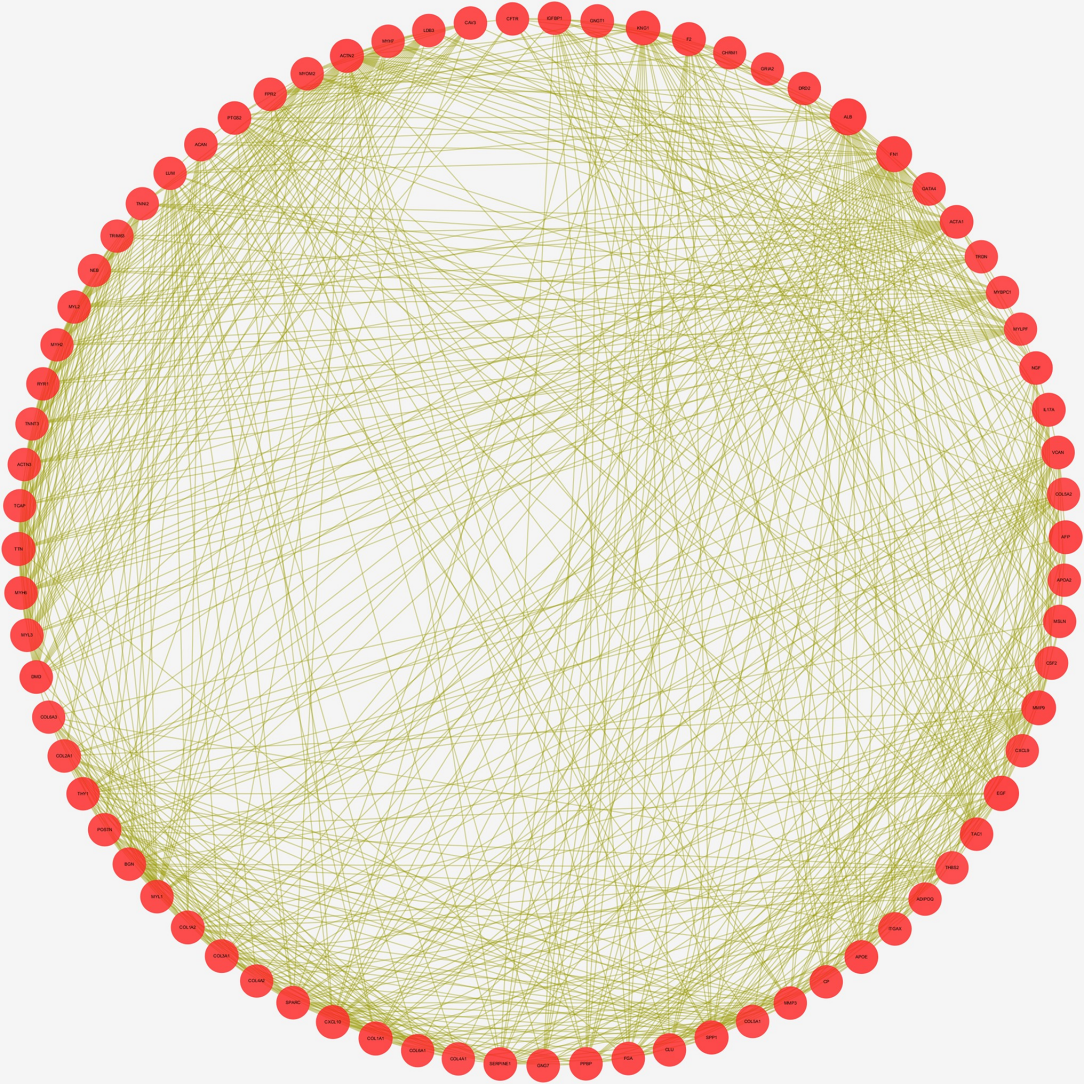
PPI network of DEmRNAs whose edges larger than 50. The size of nodes represents number of edges.

Based on DEmRNAs, 12872 pairs of mRNA interaction relationship were identified using the STRING database with scores of >0.4. Genes, whose number of edges were larger than 50, were employed in PPI network (S3 Table). 77 mRNAs were therefore selected to construct PPI network (Fig 3) using Cytoscape. The gene with the highest degree was ALB (Degree = 217), and ITGAX was the gene with the highest combined score (Combined score = 0.932).

### 4. Prognostic overall survival assessment of DEmRNAs, DEmiRNAs, and DElncRNAs

**Fig 4:**
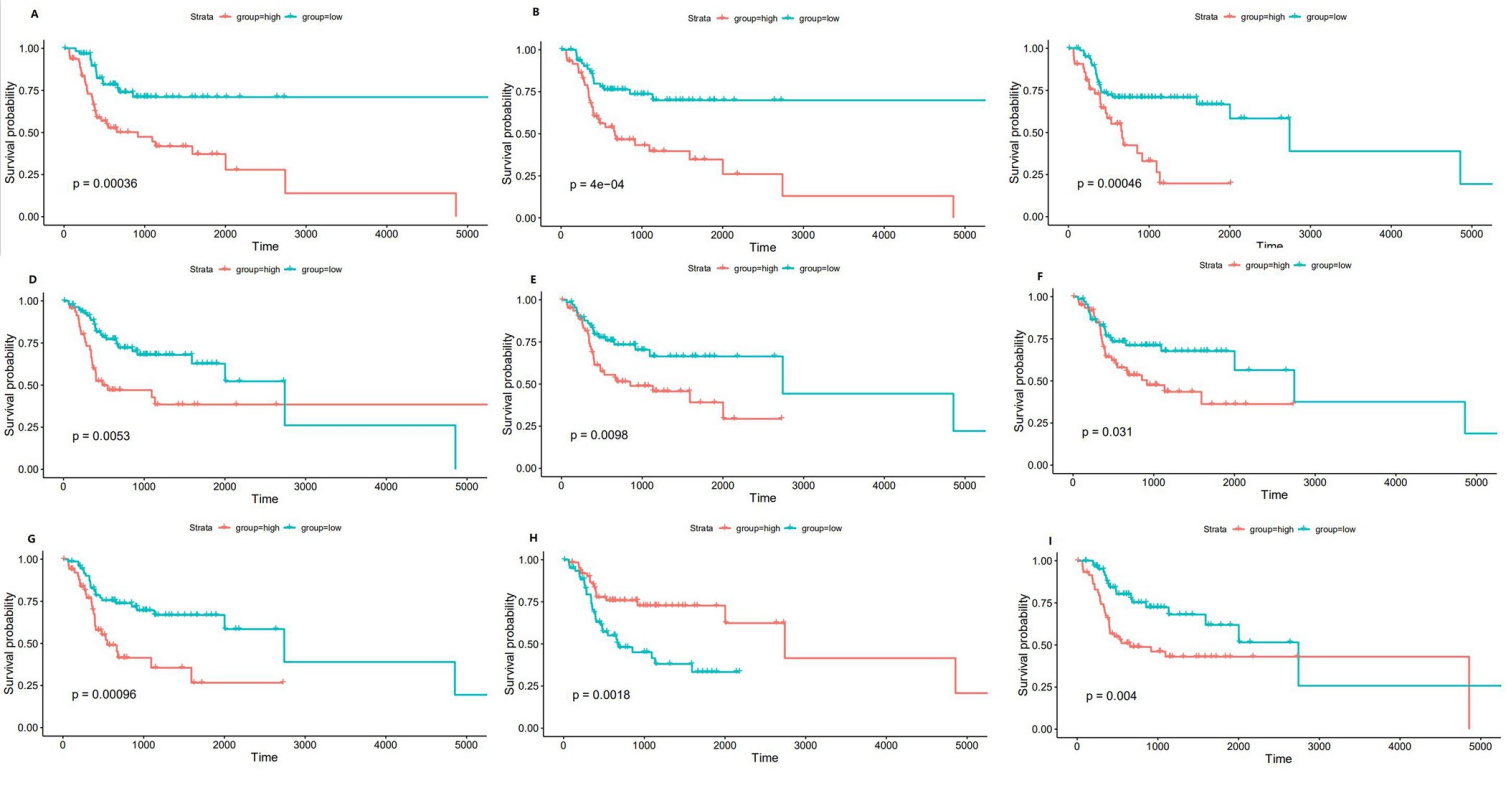
Kaplan-Meier survival plots of the top 3 prognostic RNAs; (A) NAGSKM-plot for median threshold; (B) SOHLH1 KM-plot for median threshold; (C) ETNPPL KM-plot for median threshold; (D) hsa-miR-1229-3p KM-plot for median threshold; (E) hsa-miR-654-3p KM-plot for median threshold; (F) hsa-miR-377-5p KM-plot for median threshold; (G) AL359851.1 KM-plot for median threshold; (H) LINC02560 KM-plot for median threshold; (I) AC009226.1 KM-plot for median threshold.

The survival analysis was performed to evaluate the prognostic signatures of DEmRNAs, DEmiRNAs, and DElncRNAs. Setting P<0.05 as a cutoff, a total of 154 DEmRNAs, 6 DEmiRNAs, 76 DElncRNAs were significantly related to overall survival. Among these potential prognostic biomarkers, a low expression of NAGS, which was the gene the most significantly associated with survival (P = 0.00036), was positively related to OS. For DEmiRNAs, a low expression of hsa-miR-1229-3p linked the most significantly to a high OS(P = 0.0053). However, a low expression of AL359851.1 the most significantly suggested a longer survival time, with a P = 0.00096 in DElncRNAs. The 3 biomarkers that were the most significantly related of each type of DERNAs were presented in Fig 4. All survival analysis results were given in S4 Table.

### 5. CeRNAs regulatory network construction

**Fig 5:**
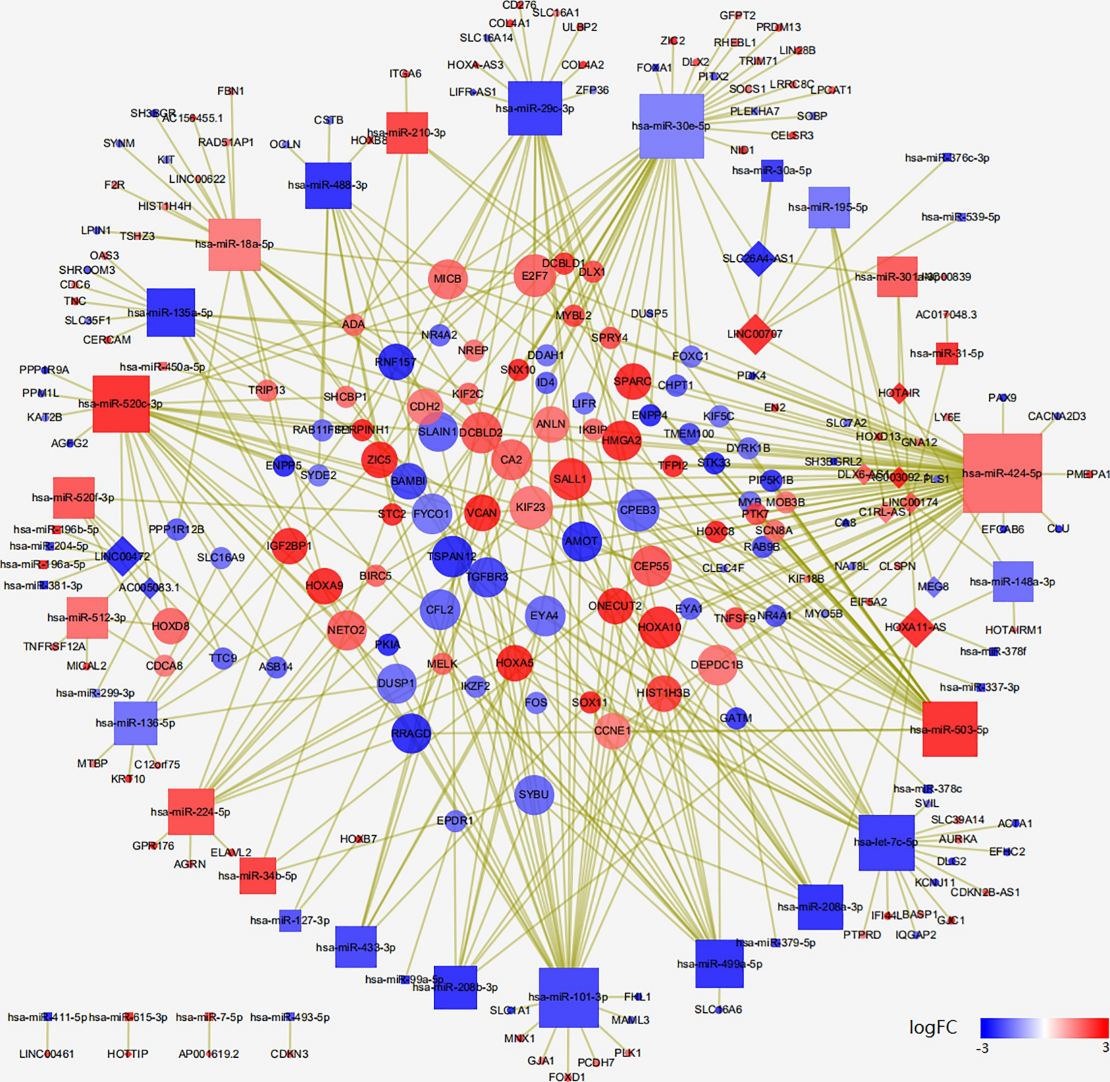
The ceRNA network of lncRNA-miRNA-mRNA in TSCC. Diamond represent lncRNA, rounded rectangles indicate miRNA, and ellipses indicate mRNA. The size of nodes represents number of edges. Red nodes indicate up-regulation while blue nodes indicate down-regulation. Different shades of color represent different levels of expression.

StarBase v3.0 was used to match DEmiRNAs and DEmRNAs or DElncRNAs. A total of 358 pairs of DEmiRNA-DEmRNA interactions (S5 Table) combined by 26 DEmiRNAs and 185 DEmRNAs were successfully predicted. In miRNA-ncRNA interaction analysis, 23 DEmiRNAs and 22 DElncRNAs were involved in 44 pairs of DEmiRNA-DElncRNA interactions (S6 Table). All the interactive pairs were integrated to construct the lncRNA-miRNA-mRNA ceRNA network (Fig 5), which contained 249 nodes and 409 edges, using cytoscape software.

To identify critical DERNAs that played an important part in both biological process and prognosis, we screened prognostic DERNAs that were involved in ceRNA network. Finally, we obtained 15 DEmRNAs(SLC16A1, E2F7, SCN8A, ZIC2, NR4A1, CLU, GFPT2, CEP55, PLK1, SYBU, STC2, DEPDC1B, MICB, LIFR, BIRC5), 1DEmiRNA(hsa-miR-337-3p) and 2 DElncRNAs(AC017048.3, AC156455.1) l. The result suggested that these RNAs were particularly crucial for the occurrence, development and prognosis of TSCC.

### 6. Validation of expressions of crucial DEmRNAs, DE-miRNAs, and DE-lncRNAs

To further confirm the biological function and prognostic value of the DEmRNAs, DE-miRNAs and DE-lncRNAs, we used GEO2R to analyze 3 GEO data (GSE30784, GSE13601 and GSE28100). SYBU, AC017048.3 and LOC100506691 were demonstrated to be non-differentially expressed in none of the mRNA microarrays. However, others were validated in at least one data (Table 2).

**Table 2:** Validation of prognostic RNAs involved in ceRNA network.

Symbol		
mRNA	GSE30784	GSE13601
SCN8A	T	T
E2F7	T	F
SLC16A1	T	T
SYBU	F	F
GFPT2	T	T
LIFR	T	T
MICB	T	T
PLK1	T	T
CEP55	F	T
STC2	T	T
BIRC5	T	T
ZIC2	T	T
DEPDC1B	T	F
NR4A1	F	T
CLU	T	T
	GSE28100	
MiR-337-3p	T	

T: True; F: False

### 7. CNV methylation and SNV analysis of hub genes

As 15 DEmRNAs mentioned above had been demonstrated as playing an important role in the development and prognosis of TSCC, we aimed to find the underlying regulatory mechanism. Methylation analysis did not show any gene whose methylation was negatively correlated with its gene expression level, by using cor package of R. However, the expression of GFPT2(Pearson's correlation coefficient = 0.436363) was possibly to be positively correlated with its CNV. SNV analysis of the most significant prognostic gene, NAGS, identified FAT1(p = 0.039) as a potential SNV locus that regulate the gene expression of NAGS.

### 8. Molecular subtypes of TSCC classification

**Fig 6:**
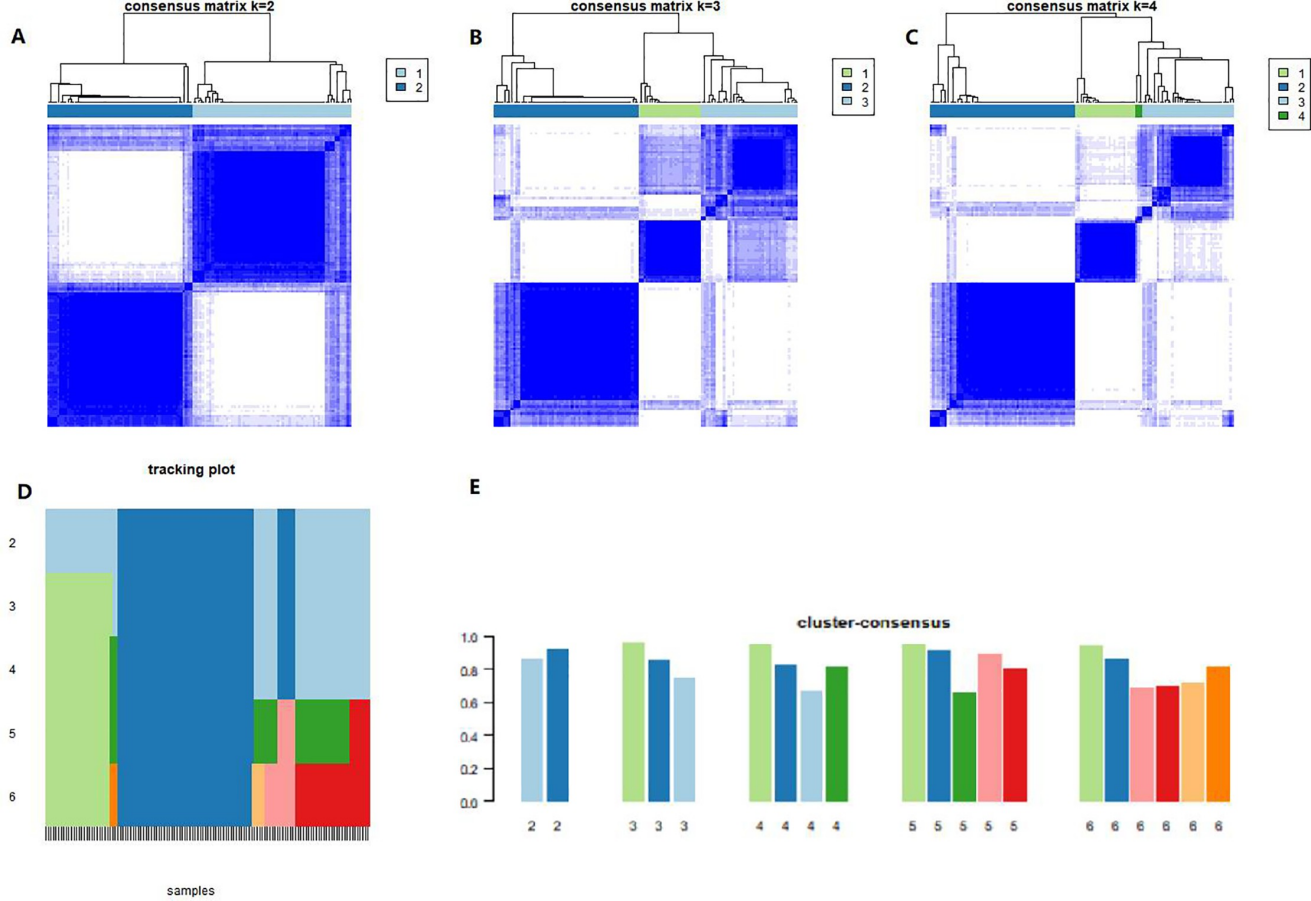
Heatmaps of the consensus matrices for k = 2(A), 3(B) and 4(C). Cluster-Consensus Plot shows the cluster-consensus value of clusters at each k. The item tracking plot(D) showed cluster assignments were stable at k = 2. Cluster-Consensus Plot(E) at k = 2 indicated reasonably high CLC among the clusters.

As DERNAs actively participated in TSCC onset and development, we speculated if there were some intrinsic gene expression subtypes related to different prognoses in TSCC. The 2000 most variable genes were employed by variance filtering (MAD method). The output of ConsensusClusterPlus showed k (2 to 6) clusters (Fig 6), and we calculated cluster-consensus and item-consensus results, which were visualized in Fig 6. All samples were successfully categorized into 2 subtypes in terms of the most stable k value, and such a process resulted in the removal of 66 out of 126 samples from subtype A and 60 out of 126 samples from subtype B.

### 9. Clinical characteristics description and prognostic assessment

**Fig 7:**
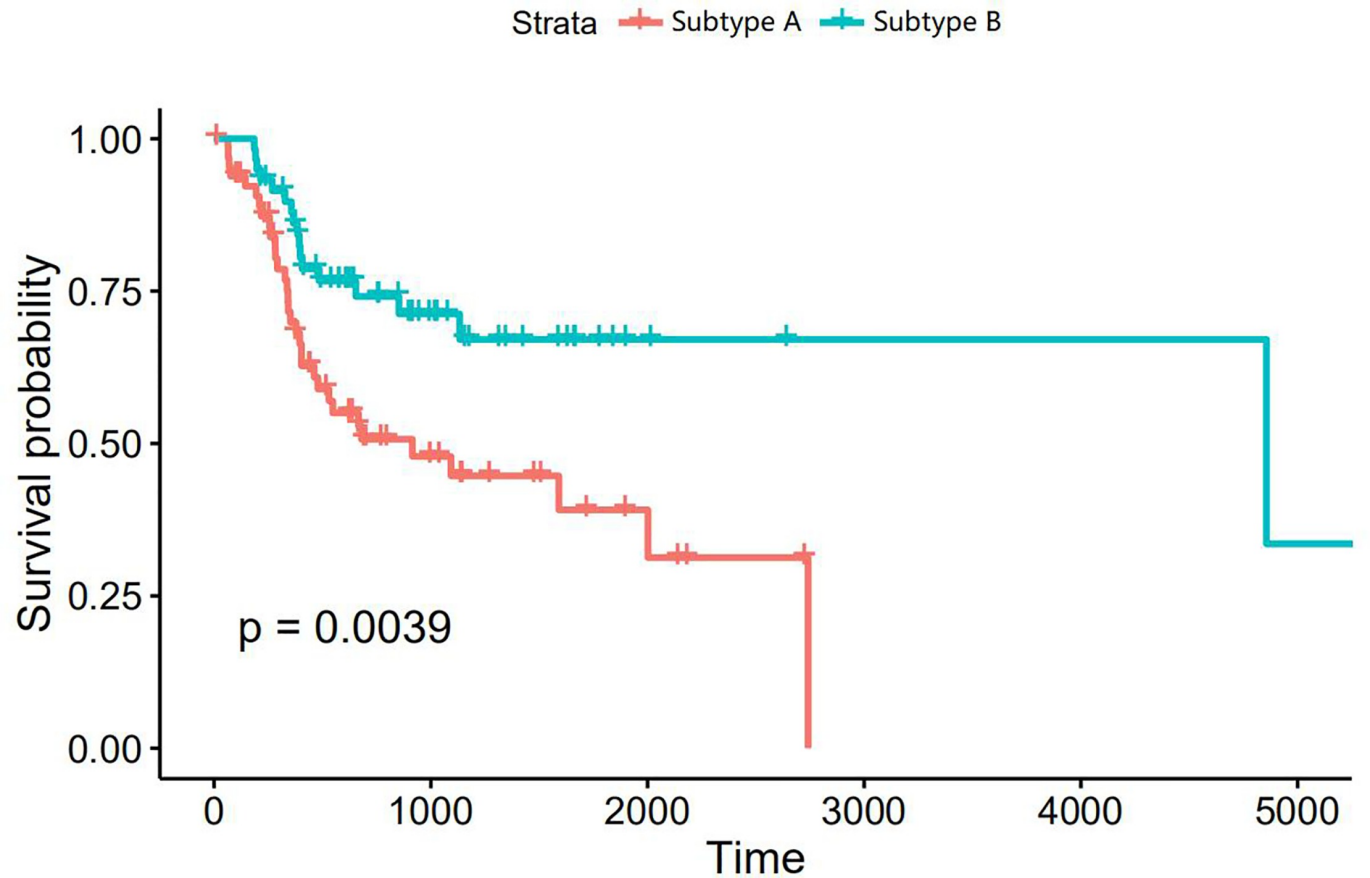
KM plot of 2 molecular subtypes of TSCC.

The clinical characteristics was described, and the results (Table 3) revealed no correlation of TSCC molecular subtype with clinical features such as gender, onset age, pathologic N or T stage, clinical stage and smoking. Noticeably, the patients in group A had a longer drink history(p = 0.024) and more alcohol consumption per day(p = 0.006). Kaplan-Meier survival curve in two subtypes (Fig 7) indicated that subtype B was significantly associated with a better overall survival(p = 0.0039).

**Table 3:** Clinical features of molecular subtypes.

Subtype	1	2	P Value
Pathologic N stage			0.697
N0	34(51.5%)	30(50.0%)	
NX	1(1.5%)	2(3.3%)	
N1	11(16.7%)	13(21.7%)	
N2	20(30.3%)	15(25%)	
Pathologic T stage			0.137
TX	1(1.5%)	1(1.7%)	
T1	3(4.5%)	8(13.3%)	
T2	27(40.9%)	23(38.3%)	
T3	21(31.8%)	22(36.7%)	
T4	14(21.2%)	6(10.0%)	
Clinical stage			0.193
1	2(3.0%)	7(11.7%)	
2	20(30.3%)	15(25%)	
3	16(24.2%)	18(30%)	
4	27(40.9%)	19(31.7%)	
x	1(1.5%)	1(1.67%)	
Smoking			0.131
Never1	19(28.8%)	25(41.7%)	
Former2	25(37.9%)	13(21.7%)	
Current3	6(9.1%)	10(16.7%)	
4	14(21.2%)	12(20%)	
Missing	2(3.0%)	0	
Smoking years			0.053
Never	19(28.8%)	25(41.7%)	
1~20	2(3.0%)	6(10%)	
20~30	4(6.1%)	5(8.3%)	
30~	29(43.9%)	19(31.7%)	
Mising	12(18.2%)	5(8.3%)	
Alcohol			0.024
Yes	49(74.2%)	33(55.0%)	
No	17(25.8%)	27(45.0%)	
Alcohol consumption per day			0.006
0	22(33.3%)	34(56.7%)	
1~5	24(36.4%)	10(16.7%)	
5~	2(3.0%)	6(10.0%)	
missing	18(27.3%)	10(16.7%)	
Gender			0.254
Male	46(69.7%)	36(60.0%)	
Female	20(30.3%)	24(40.0%)	
Onset age			0.55
15~30	2(3.0%)	5(8.3%)	
30~50	12(18.2%)	13(21.7%)	
50~70	42(63.6%)	34(56.7%)	
70~	10(15.2%)	8(13.3%)	
Pathological nodal extracapsular spread			0.033
Yes	14(21.2%)	10(16.7%)	
No	35(53.0%)	44(73.3%)	
Unknown	17(25.8%)	6(10%)	

### 10. Identification of differential expression of mRNAs, miRNAs, lncRNAs between different subtypes of TSCC overlapping with prognostic DERNAs and functional enrichment analysis of GO and KEGG

**Fig 8:**
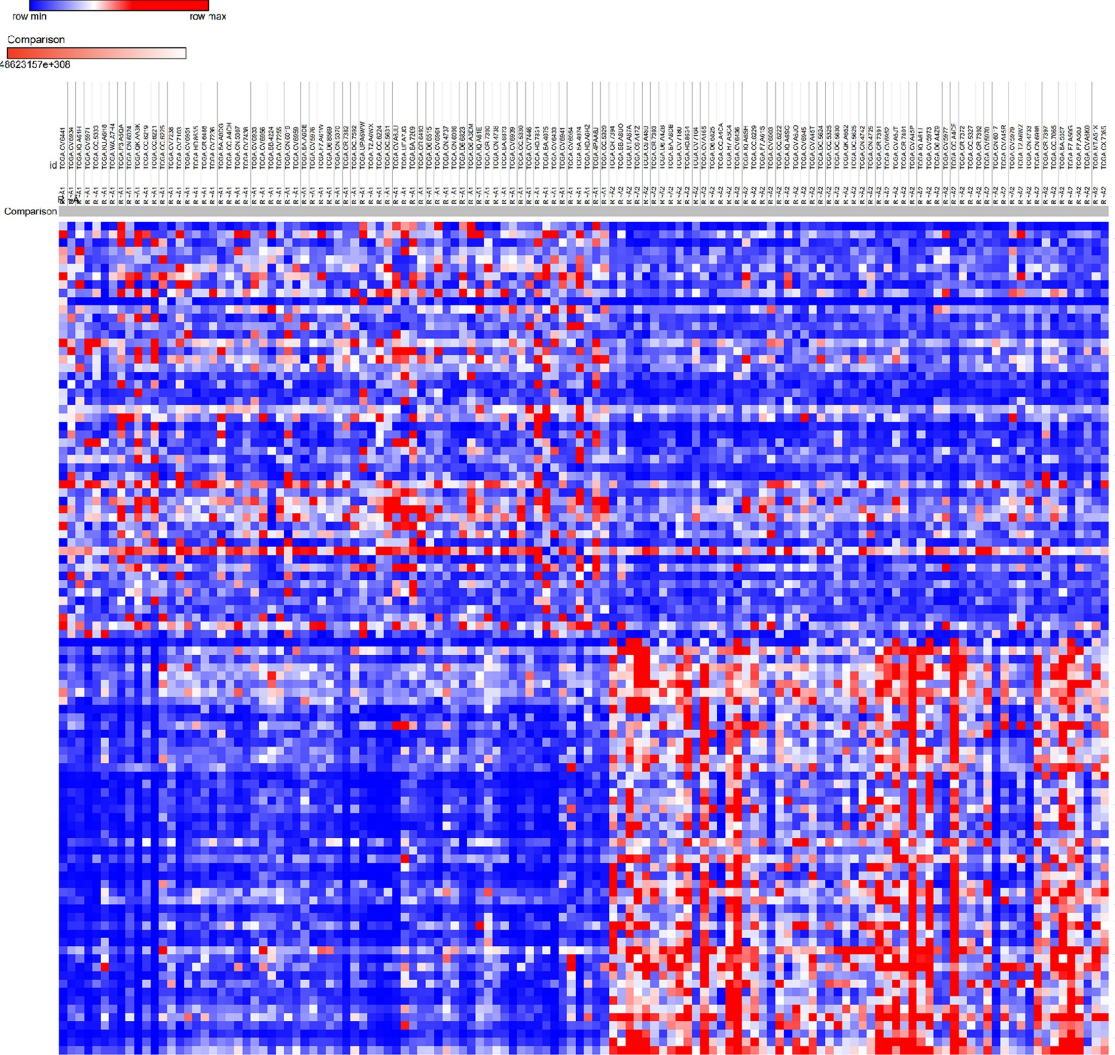
Heatmap of DEmRNAs between 2 molecular subtypes of TSCC.

**Fig 9:**
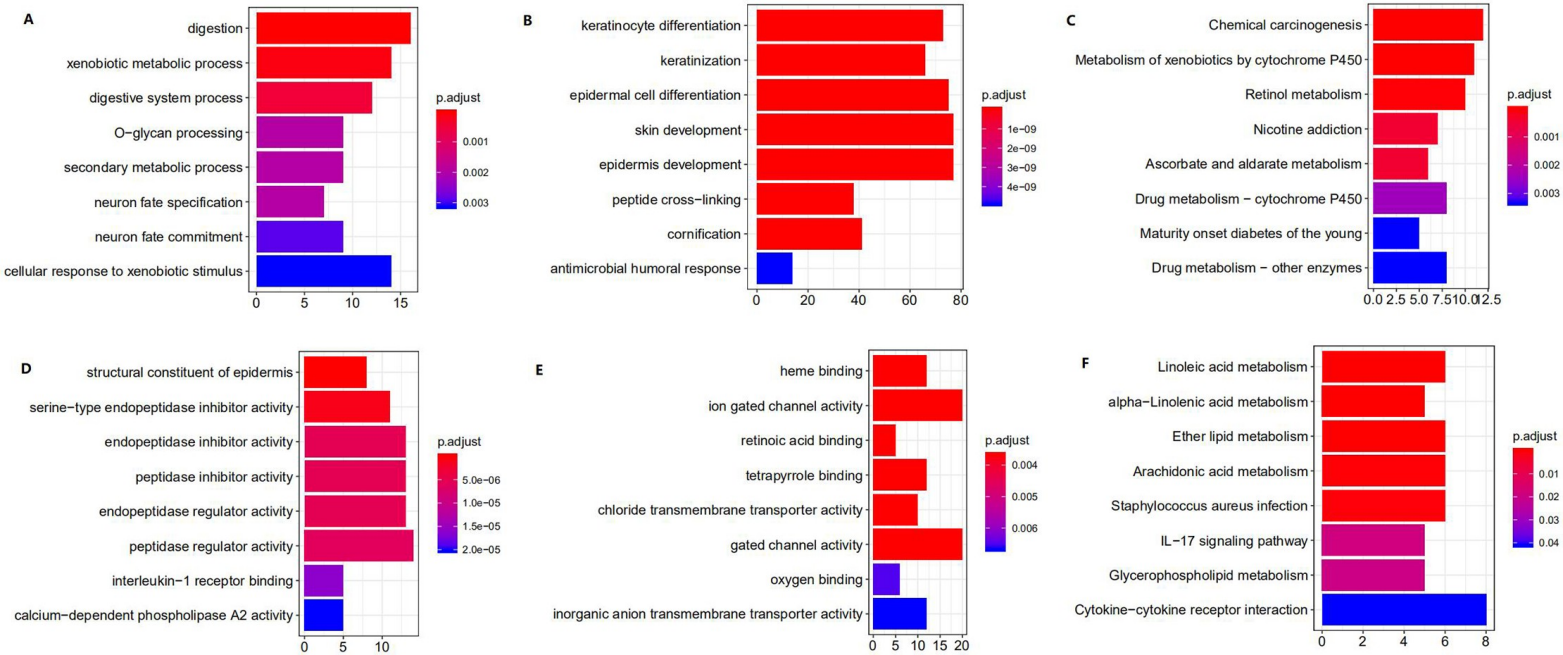
The top 10 enrichment scores in KEGG pathway and GO enrichment analysis of the DEmRNA between two subtypes. (A)BP of up-regulatory mRNA in subtype A; (B) BP of up-regulatory mRNA in subtype B; (C) MF of up-regulatory mRNA in subtype A; (D)MF of up-regulatory mRNA in subtype B (E) KEGG pathway of up-regulatory mRNA in subtype A; (F) KEGG pathway of up-regulatory mRNA in subtype B.

After performing DESeq2 package, 715 DEmRNAs, 7 DEmiRNAs and 521 DElncRNAs were screened. Top 10 DEmRNAs between two subtypes were list in Table 4. Morpheus website was applied for plotting the heatmap of DEmRNAs (Fig 8). The 3 most upregulated genes were BPIFB2, CTCFL and NTS in subtype A, while DEFB4B, CRNN and MUC21 were the 3 most up-regulated genes in subtype B. DERNAs, 25 mRNAs, 5lncRNAs and 0 miRNA were found to overlapped with prognostic RNAs mentioned above (Table 5). Further enrichment analysis (S7 Table) showed that upregulated genes in subtype A were highly enriched not only in metabolic process, digestion and multicellular organismal homeostasis in GO, but also in chemical carcinogenesis, metabolism by cytochrome P450 and in nicotine addiction in KEGG (Fig 9). However, upregulated genes in group B (S8 Table) was involved in GO including keratinocyte or epidermal cell differentiation, skin or development, peptide cross-linking and cell killing and KEGG, including lipid or acid metabolism and IL-17 signal pathway. Therefore, we termed these two subtypes ‘basal’ and ‘differentiated’ in terms of their particular differential expression gene functions and literature reviews.

**Table 4:** Top 10 DEmRNAs between two molecular subtypes.

Symbol	Log2FoldChange	Pvalue	High expression in which subtype
BPIFB2	-6.12855	1.75E-10	A
DEFB4B	5.844115	2.73E-21	B
CTCFL	-5.78405	1.47E-16	A
NTS	-5.5462	5.91E-27	A
BPIFB1	-5.41866	6.25E-20	A
CRNN	5.009013	2.23E-22	B
CYP26A1	-4.70985	3.56E-16	A
MUC5B	-4.47367	7.41E-20	A
MUC21	4.343534	6.98E-15	B
MUC2	-4.32957	2.87E-25	A

**Table 5:** DERNAs between two subtypes related to prognosis.

Symbol	logFC	P value of survival analysis
SOHLH1	-4.108109034	0.000401198
TGM5	1.856189234	0.001104156
ODF4	1.83110008	0.002145965
SPINK7	4.11976683	0.004824705
FUT6	1.522467309	0.00743154
RNF224	1.914806608	0.008302427
CNFN	2.680331441	0.010640736
PNCK	-2.685197192	0.016092106
PCP4	-1.814741921	0.017260067
FAM25A	2.833964095	0.01735488
HPN	-2.256573085	0.019961787
SPRR2E	2.816019954	0.020093855
CLDN17	2.912792033	0.020322063
S100A9	2.13252753	0.024516409
SLC2A14	-1.683662504	0.026262725
LEXM	2.217909192	0.028836265
CRCT1	2.901957091	0.028858706
TGM7	2.447755921	0.030069596
MAL	3.924051739	0.033186621
CCDC187	-2.418284944	0.037448993
SPRR2A	2.669076678	0.037724674
CCDC155	-2.442240546	0.047564285
C9orf152	-2.351492462	0.048737265
SLC9A2	-2.31320548	0.049015
SPINK6	3.219429317	0.049538758
LINC02560	1.76588842	0.001813109
LINC02028	2.121778242	0.016551484
LOC349160	-1.806825885	0.023342443
LINC02487	1.783316228	0.032572701
LOC101927503	-1.858832402	0.04787

## Discussion

TSCC, which is the most common kind of OSCC, is generally characterized by a notably aggressive biological behavior and a poor survival. It is urgent to detect the underlying genetic pathogenesis and to find reliable therapeutic targets and prognostic biomarkers for TSCC in order to improve patients’ clinical outcome. In this study, we used TCGA database to identify mRNAs, miRNAs, lncRNA with differential expressions between TSCC and normal control tissues. To further understand the DERNAs, GO and KEGG analysis were performed subsequently. The results of GO analysis showed that extracellular matrix organization, extracellular structure organization, muscle contraction, regulation of ion transmembrane transport and muscle system process were involved in the biological process of TSCC initiation, which was different from other GO types of OSCC [16,17]. This suggested TSCC might be a special type of OSCC, which might be resulted from particular anatomical and histological structure of the tongue. Moreover, the enriched KEGG pathway was found to be involved in cytokine-cytokine receptor interaction, PI3K-Akt signaling pathway, HPV infection, alcoholism, focal adhesion, MAPK signaling pathway and cAMP signaling pathway, which was consistent with findings from other previous studies on the tumorigenesis of TSCC [18,19]. We next constructed the PPI network with DEmRNAs and obtained the hub genes with the highest degree including ALB, FN1, EGF, MM9, KNG1, COL1A1, SPP1, ACTN2, most of which were reported to play a critical role in carcinogenesis and tumor progression [20–23].

The ceRNA network was established by integrating all DERNAs. A total of 15 prognostic DEmRNAs (SLC16A1, E2F7, SCN8A, ZIC2, NR4A1, CLU, GFPT2, CEP55, PLK1, SYBU, STC2, DEPDC1B, MICB, LIFR, BIRC5), 1 DEmiRNA(hsa-miR-337-3p) and 2 DElncRNAs (AC017048.3, AC156455.1) were involved in ceRNA network. We investigated the underlying mechanism of mRNA with differential expression, and apart from GFPT2, no correlation was identified between CNV or methylation and gene expression, which provided foundation for intensive study in future. SNV analysis of NAGS (the most significant prognostic gene) indicated that somatic mutation in FAT1(atypical cadherin 1) may be associated with the dysregulation of NAGS. According to Pickering, FAT1 was mutated in 30% of OSCC samples, which was also the highest frequency of mutation in HNSCC [24,25]. Researchers also suggested that FAT1 inhibited migration and invasion in OSCC and some other types cancers [26,27]. In this study, FAT1 mutation was shown to be related to differential expression of NAGS, which resulted in a remarkable variation of overall survival. All these results suggested that FAT1 could be used as a novel candidate tumor suppressor or prognostic predictor.

SLC16A1(solute carrier family 16 member 1), alternatively known as MCT, is the most significant prognostic DEmRNA employed in ceRNA network and might have the potential to be an important biomarker of survival prediction and therapeutic target in TSCC. Recent studies have confirmed our speculation, Voelxen found that tumor samples from OSCC patients had significantly elevated relative expression levels of MCT [28]. Susana et al. suggested that MCT might be a prognostic molecule of OSCC [29]. E2F7(transcription factor 7), targeted by MIR424, miR-3666 and miR-26a, is highly expressed in multiple tumors [30,31] such as endometrial carcinoma, breast cancer, colorectal cancer and glioma, indicating that E2F7 might constitute a potential therapeutic target. The overexpression of E2F7 was also detected in HNSCC [32]. It was also found that Sphk1-dependent activation of AKT mediated E2F7-induced doxorubicin resistance, which might be similar to the mechanism of progression in TSCC. SCN8A (sodium voltage-gated channel alpha subunit 8), which is related to different types of cancers [33,34] including colorectal cancer, prostate cancer, breast and some other types of cancer, emerges as an aggressive oncogene and could cause poor prognosis [35]. ZIC2(Zic family member 2) has been reported to be crucial to the progression of cancer [36–38] such as hepatocellular carcinoma, epithelial ovarian tumor, osteosarcoma and OSCC. ZIC2 could activated the PI3K/AKT signal pathway, which played a vital role in TSCC, as it promotes viability, migration, and invasion of cancer cells. Sakuma [38] et al. detected that a significant up-regulation of ZIC2 in OSCC samples was related to a poor survival rate. NR4A1(nuclear receptor subfamily 4 group A member 1) was validated to be targeted by miR-377 [39] and PCH4 [40] and act as a tumor promoter in OSCC. Besides, Liu and Chen [41,42] discovered that NR4A1 could be a critical general regulator in the induction of T cell dysfunction and inhibit NR4A, thus, NR4A1 was seen as promising in cancer immunotherapy. The prognostic DEmiRNA, hsa-miR-337-3p targeting one DEmRNA (AMOT, a witnessed oncogene in OSCC [43]), has been implicated to target AMOT, HOXB7 [44], MMP-14 [45], PTEN [46] and acted as a tumor suppressor through sponging oncogene. Loss of miR-337-3p expression was associated with the development of cancer [47]. Most of these DERNAs were demonstrated to participate in carcinogenesis and progression in existing studies. However, the underlying functions of the 2 prognostic DElncRNAs in cancer remain poorly understood.

To further explore the possible genetic classification of TSCC, we identified 2 molecular subtypes in TSCC and termed these 2 subtypes basal and differentiated, according to gene function and Pickering’s work on integrative genomic analysis of OSCC [48]. We integrated the clinical data of the 2 subtypes, interestingly, we found that basal tumors were highly associated with alcohol consumption but a poor clinical outcome. Moreover, the basal tumors were likely to come from patients with a smoking habit. All these clinical features suggested that alcohol and tobacco participate in critical progression of TSCC, therefore leading to a worse prognosis. In addition, we obtained 715 DEmRNAs, 7 DEmiRNAs, 521DElncRNAs. The functional analysis results suggested that basal tumor was expected to be related to metabolic process, chemical carcinogenesis and nicotine addiction, which could explain its different clinical manifestation. However, differentiated tumor was shown to participate in keratinocyte or epidermal cell differentiation, skin or epithelial development, peptide cross-linking, cell killing and IL-17 signal pathway, which were similar to the features characterized by a previous study on differentiated subtype [49]. These findings contributed to a better understanding on the difference of clinical characteristics and the underlying biological mechanism between different subtypes of TSCC.

Among these DERNAs, 25 mRNAs and 5lncRNAs were significantly correlated to prognostic RNAs and had a potential to act as predicted biomarkers to evaluate prognosis. These findings are mostly consistent with the results of previous studies. ODF4 (outer dense fiber of sperm tails 4) has been reported to have a high expression [50] in breast cancer, prostate cancer, basal cell carcinoma and chronic myeloid lymphoma. Afsharpad [51] determined ODF4 expression in urinary exfoliated cells, cancerous tissue and tumor-free tissue to confirm its diagnostic and surveillance potential. SPINK7(serine peptidase inhibitor, Kazal type 7 (putative)) targeted by miR-1322[52] by inhibiting invasion of cancer cells via the urokinase-type plasmin activator receptor 1 integrin pathway or by upregulating p53 expression was considered as a tumor suppressor in multiple types cancers [53–55]. Li et al [56] validated the predictive value of SPINK7 in noninvasive early detection of gastric cancer. Therefore, it is possible to predict prognosis of TSCC patients by detecting SPINK7 expression in saliva. The dysregulation of LINC02487, alternatively known as LOC441178, was detected by transcriptome analysis and verified by RT-PCR in OSCC [57]. Xu [58] et al found that LINC02487 could sponge coiled-coil-containing protein kinase 1 (ROCK1) in OSCC, and that OSCC patients with a high expression of LINC02487 would have a longer survival time, which was in accordance with our result and also suggested that LINC02487 could be a new therapeutic target and prognostic indicator for TSCC. To the best of our knowledge, no relevant studies found that LINC02028, LOC101927503, LOC349160 and LINC02560 existed in cancer.

With the advance of RNA sequencing and bioinformatic analysis, a large amount of genomic data become available but requires to be decoded. In our study, we found that 15 DEmRNAs, 1 DEmiRNA, 2 DElncRNAs were related to prognosis by constructing ceRNA network of TSCC. Most of them were found to act as oncogenes or tumor suppressors in many types of cancer, and the analysis of GO and KEGG pathway and PPI network further confirmed their function. Combining the ceRNA network theory, we considered that these DERNAs played a critical role in the occurrence of TSCC and could be utilized as biomarkers for target therapy. Additionally, we firstly confirmed 2 molecular subtypes in TSCC using data from TCGA by clustering analysis, and such an effort helped comprehend the intrinsic classification of TSCC and specific biological process of each subtype. We correlated DEmRNAs between basal and differentiated tumor with prognosis, and 25 mRNAs and 5 lncRNAs were identified to be associated with prognosis. Evidence suggested that these critical molecules not only participated in the initiation, but also in the development of TSCC and helped deciding prognosis and therapy decision. Advances and limitations of this study should also be acknowledged. It should be noticed that we integrated several categories of genetic data to bring out the importance of these selected molecules at different regulatory levels, which has hardly been done in previous studies. However, the data from TCGA only included American samples, which could not represent the whole existing conditions and as the regulatory pathway of molecular is very intricate, what we found in the study may be the tip of an iceberg. Thus, the mechanism of TSCC or even in all types of cancer still has an immense potential to be discovered, and further clinical trials and molecular experiments are required to verify our results.

## Conclusion

The TCGA-based comprehensive genomic analyses successfully established a ceRNA network of TSCC and defined 2 intrinsic molecular subtypes of TSCC. Furthermore, we demonstrated key molecules involved in carcinogenesis and progression by integrating results of analyses of ceRNA network, overall survival and subtype classification. These discoveries provided a novel genetic landscape and the foundation for prognostic prediction or for more effective treatment, in which key genes are checked and targeted by specific inhibitors or promotors

## Supporting information

S1 Table GO analyses of DEmRNAs. (DOCX)

S2 Table KEGG pathways of DEmRNAs. (DOCX)

S3 Table PPI network nodes of DEmRNAs. (DOCX)

S4 Table Survival analyses of DERNAs. (DOCX)

S5 Table 358 pairs of DEmiRNA-DEmRNA interactions. (DOCX)

S6 Table 44 pairs of DEmiRNA-DElncRNA interactions. (DOCX)

S7 Table Functional enrichment analyses of GO and KEGG in subtype A. (DOCX)

S8 Table Functional enrichment analyses of GO and KEGG in subtype B. (DOCX)
