# Correction: TCGA based integrated genomic analyses of ceRNA network and novel subtypes revealing potential biomarkers for the prognosis and target therapy of tongue squamous cell carcinoma

**DOI:** 10.1371/journal.pone.0218987

**Published:** 2019-06-20

**Authors:** The PLOS ONE Staff

There are errors in the Funding statement. The publisher apologizes for the errors. The correct Funding statement is as follows: This study was supported by the Fundamental Research Funds for the Central Universities of Central South University (1053320182836). The funder had no role in study design, data collection and analysis, decision to publish, or preparation of the manuscript.
