# Supplementary material for: TCGA based integrated genomic analyses of ceRNA network and novel subtypes revealing potential biomarkers for the prognosis and target therapy of tongue squamous cell carcinoma
Source: PLoS One. 2019 May 29;14(5):e0216834. doi: 10.1371/journal.pone.0216834 (PMC6541473; doi:10.1371/journal.pone.0216834)
Supplement: S1 Table — (DOCX) [file pone.0216834.s001.docx]

**S1 Table: GO analyses of DEmRNAs**

| GO category | ID | Description | pvalue | Count |
| --- | --- | --- | --- | --- |
| biological process | GO:0030198 | extracellular matrix organization | 1.20E-33 | 141 |
| biological process | GO:0043062 | extracellular structure organization | 2.60E-32 | 152 |
| biological process | GO:0006936 | muscle contraction | 2.55E-20 | 110 |
| biological process | GO:0034765 | regulation of ion transmembrane transport | 1.25E-16 | 125 |
| biological process | GO:0003012 | muscle system process | 2.28E-16 | 124 |
| biological process | GO:0030049 | muscle filament sliding | 2.31E-16 | 27 |
| biological process | GO:0033275 | actin-myosin filament sliding | 2.31E-16 | 27 |
| biological process | GO:0061448 | connective tissue development | 2.57E-15 | 83 |
| biological process | GO:0070252 | actin-mediated cell contraction | 5.15E-15 | 48 |
| biological process | GO:0048705 | skeletal system morphogenesis | 5.97E-15 | 76 |
| biological process | GO:0070268 | cornification | 6.68E-14 | 49 |
| biological process | GO:1904062 | regulation of cation transmembrane transport | 1.15E-13 | 90 |
| biological process | GO:0043588 | skin development | 1.41E-13 | 118 |
| biological process | GO:0051216 | cartilage development | 3.80E-13 | 67 |
| biological process | GO:0043270 | positive regulation of ion transport | 1.27E-12 | 77 |
| biological process | GO:0030048 | actin filament-based movement | 1.57E-12 | 50 |
| biological process | GO:0031424 | keratinization | 3.47E-12 | 77 |
| biological process | GO:0032963 | collagen metabolic process | 3.60E-12 | 44 |
| biological process | GO:0018149 | peptide cross-linking | 3.93E-12 | 30 |
| biological process | GO:0048706 | embryonic skeletal system development | 4.61E-12 | 47 |
| biological process | GO:0032412 | regulation of ion transmembrane transporter activity | 5.43E-12 | 71 |
| biological process | GO:0032409 | regulation of transporter activity | 6.90E-12 | 75 |
| biological process | GO:0002062 | chondrocyte differentiation | 7.01E-12 | 44 |
| biological process | GO:0060537 | muscle tissue development | 9.61E-12 | 102 |
| biological process | GO:0030216 | keratinocyte differentiation | 1.01E-11 | 90 |
| biological process | GO:0003002 | regionalization | 1.62E-11 | 92 |
| biological process | GO:0030239 | myofibril assembly | 1.99E-11 | 31 |
| biological process | GO:0022898 | regulation of transmembrane transporter activity | 2.24E-11 | 71 |
| biological process | GO:0014706 | striated muscle tissue development | 2.65E-11 | 98 |
| biological process | GO:0010959 | regulation of metal ion transport | 2.77E-11 | 99 |
| biological process | GO:0007389 | pattern specification process | 2.83E-11 | 108 |
| biological process | GO:0055074 | calcium ion homeostasis | 7.70E-11 | 111 |
| biological process | GO:0009952 | anterior/posterior pattern specification | 1.01E-10 | 64 |
| biological process | GO:1904064 | positive regulation of cation transmembrane transport | 1.79E-10 | 46 |
| biological process | GO:0006941 | striated muscle contraction | 1.86E-10 | 53 |
| biological process | GO:0007517 | muscle organ development | 2.42E-10 | 100 |
| biological process | GO:0034767 | positive regulation of ion transmembrane transport | 3.39E-10 | 48 |
| biological process | GO:0009913 | epidermal cell differentiation | 3.40E-10 | 97 |
| biological process | GO:0034764 | positive regulation of transmembrane transport | 3.56E-10 | 57 |
| biological process | GO:0007492 | endoderm development | 3.56E-10 | 33 |
| biological process | GO:0060538 | skeletal muscle organ development | 3.72E-10 | 54 |
| biological process | GO:0015711 | organic anion transport | 4.52E-10 | 108 |
| biological process | GO:0030199 | collagen fibril organization | 6.13E-10 | 30 |
| biological process | GO:2001257 | regulation of cation channel activity | 7.40E-10 | 51 |
| biological process | GO:0060047 | heart contraction | 9.04E-10 | 66 |
| biological process | GO:0048562 | embryonic organ morphogenesis | 9.30E-10 | 76 |
| biological process | GO:0051146 | striated muscle cell differentiation | 9.30E-10 | 76 |
| biological process | GO:0003015 | heart process | 1.43E-09 | 67 |
| biological process | GO:0042391 | regulation of membrane potential | 2.52E-09 | 101 |
| biological process | GO:0006874 | cellular calcium ion homeostasis | 2.84E-09 | 104 |
| cellular component | GO:0062023 | collagen-containing extracellular matrix | 7.03E-31 | 129 |
| cellular component | GO:0043292 | contractile fiber | 3.87E-29 | 99 |
| cellular component | GO:0030016 | myofibril | 4.11E-29 | 96 |
| cellular component | GO:0044449 | contractile fiber part | 4.13E-29 | 95 |
| cellular component | GO:0030017 | sarcomere | 4.89E-28 | 89 |
| cellular component | GO:0031674 | I band | 1.46E-19 | 62 |
| cellular component | GO:0005788 | endoplasmic reticulum lumen | 1.61E-18 | 99 |
| cellular component | GO:0030018 | Z disc | 2.08E-17 | 56 |
| cellular component | GO:0005581 | collagen trimer | 9.24E-16 | 43 |
| cellular component | GO:0044420 | extracellular matrix component | 8.66E-15 | 31 |
| cellular component | GO:0042383 | sarcolemma | 4.03E-14 | 53 |
| cellular component | GO:0031672 | A band | 5.69E-14 | 26 |
| cellular component | GO:1902495 | transmembrane transporter complex | 8.86E-14 | 95 |
| cellular component | GO:1990351 | transporter complex | 3.81E-13 | 95 |
| cellular component | GO:0034702 | ion channel complex | 3.86E-13 | 89 |
| cellular component | GO:0016528 | sarcoplasm | 1.70E-11 | 33 |
| cellular component | GO:0001533 | cornified envelope | 9.02E-11 | 31 |
| cellular component | GO:0031430 | M band | 1.87E-10 | 19 |
| cellular component | GO:0016529 | sarcoplasmic reticulum | 3.11E-10 | 29 |
| cellular component | GO:0016459 | myosin complex | 4.78E-10 | 29 |
| cellular component | GO:0005604 | basement membrane | 5.04E-10 | 35 |
| cellular component | GO:0034703 | cation channel complex | 8.17E-10 | 63 |
| cellular component | GO:0098644 | complex of collagen trimers | 2.10E-09 | 16 |
| cellular component | GO:0005865 | striated muscle thin filament | 3.95E-09 | 18 |
| cellular component | GO:0036379 | myofilament | 7.69E-09 | 18 |
| cellular component | GO:0031225 | anchored component of membrane | 4.70E-08 | 51 |
| cellular component | GO:0032982 | myosin filament | 1.18E-07 | 14 |
| cellular component | GO:0016323 | basolateral plasma membrane | 1.61E-07 | 59 |
| cellular component | GO:0045177 | apical part of cell | 3.19E-07 | 87 |
| cellular component | GO:0016324 | apical plasma membrane | 3.36E-07 | 76 |
| cellular component | GO:0009897 | external side of plasma membrane | 4.82E-07 | 77 |
| cellular component | GO:0034707 | chloride channel complex | 9.76E-07 | 22 |
| cellular component | GO:0033017 | sarcoplasmic reticulum membrane | 1.30E-06 | 17 |
| cellular component | GO:0030315 | T-tubule | 1.71E-06 | 20 |
| cellular component | GO:0097060 | synaptic membrane | 2.87E-06 | 93 |
| cellular component | GO:0045211 | postsynaptic membrane | 2.99E-06 | 75 |
| cellular component | GO:0044304 | main axon | 5.63E-06 | 24 |
| cellular component | GO:0005583 | fibrillar collagen trimer | 7.33E-06 | 10 |
| cellular component | GO:0098643 | banded collagen fibril | 7.33E-06 | 10 |
| cellular component | GO:0016460 | myosin II complex | 8.45E-06 | 12 |
| cellular component | GO:0033267 | axon part | 1.59E-05 | 77 |
| cellular component | GO:0014704 | intercalated disc | 2.25E-05 | 18 |
| cellular component | GO:0005859 | muscle myosin complex | 2.75E-05 | 10 |
| cellular component | GO:0031093 | platelet alpha granule lumen | 4.92E-05 | 22 |
| cellular component | GO:0005796 | Golgi lumen | 7.39E-05 | 35 |
| cellular component | GO:0034705 | potassium channel complex | 0.000184 | 27 |
| cellular component | GO:0046658 | anchored component of plasma membrane | 0.000196 | 18 |
| cellular component | GO:0030673 | axolemma | 0.0002 | 9 |
| cellular component | GO:0034706 | sodium channel complex | 0.000212 | 10 |
| cellular component | GO:0060076 | excitatory synapse | 0.000237 | 16 |
| molecular function | GO:0005201 | extracellular matrix structural constituent | 1.64E-27 | 82 |
| molecular function | GO:0022838 | substrate-specific channel activity | 4.90E-19 | 133 |
| molecular function | GO:0005216 | ion channel activity | 6.54E-17 | 125 |
| molecular function | GO:0022836 | gated channel activity | 1.26E-13 | 102 |
| molecular function | GO:0022839 | ion gated channel activity | 5.76E-13 | 97 |
| molecular function | GO:0030020 | extracellular matrix structural constituent conferring tensile strength | 2.29E-12 | 25 |
| molecular function | GO:0005261 | cation channel activity | 1.61E-11 | 88 |
| molecular function | GO:0046873 | metal ion transmembrane transporter activity | 2.11E-11 | 113 |
| molecular function | GO:0008509 | anion transmembrane transporter activity | 3.58E-11 | 91 |
| molecular function | GO:0015077 | monovalent inorganic cation transmembrane transporter activity | 3.59E-11 | 99 |
| molecular function | GO:0005125 | cytokine activity | 3.85E-11 | 74 |
| molecular function | GO:0008307 | structural constituent of muscle | 1.41E-10 | 24 |
| molecular function | GO:0015108 | chloride transmembrane transporter activity | 1.56E-09 | 39 |
| molecular function | GO:0005539 | glycosaminoglycan binding | 2.84E-09 | 65 |
| molecular function | GO:0030414 | peptidase inhibitor activity | 3.80E-09 | 58 |
| molecular function | GO:0004252 | serine-type endopeptidase activity | 5.17E-09 | 60 |
| molecular function | GO:0005244 | voltage-gated ion channel activity | 8.17E-09 | 58 |
| molecular function | GO:0022832 | voltage-gated channel activity | 8.17E-09 | 58 |
| molecular function | GO:0015081 | sodium ion transmembrane transporter activity | 9.85E-09 | 47 |
| molecular function | GO:0004222 | metalloendopeptidase activity | 9.95E-09 | 41 |
| molecular function | GO:0008083 | growth factor activity | 1.53E-08 | 49 |
| molecular function | GO:0017171 | serine hydrolase activity | 1.70E-08 | 64 |
| molecular function | GO:0061134 | peptidase regulator activity | 1.70E-08 | 64 |
| molecular function | GO:0008237 | metallopeptidase activity | 2.71E-08 | 56 |
| molecular function | GO:0001228 | DNA-binding transcription activator activity, RNA polymerase II-specific | 2.91E-08 | 103 |
| molecular function | GO:0004866 | endopeptidase inhibitor activity | 5.16E-08 | 54 |
| molecular function | GO:0001077 | proximal promoter DNA-binding transcription activator activity, RNA polymerase II-specific | 5.22E-08 | 73 |
| molecular function | GO:0008236 | serine-type peptidase activity | 5.25E-08 | 62 |
| molecular function | GO:0005254 | chloride channel activity | 8.26E-08 | 30 |
| molecular function | GO:0061135 | endopeptidase regulator activity | 9.90E-08 | 55 |
| molecular function | GO:0051393 | alpha-actinin binding | 1.58E-07 | 17 |
| molecular function | GO:0004857 | enzyme inhibitor activity | 1.92E-07 | 92 |
| molecular function | GO:0042805 | actinin binding | 2.24E-07 | 19 |
| molecular function | GO:0008514 | organic anion transmembrane transporter activity | 2.95E-07 | 56 |
| molecular function | GO:0003779 | actin binding | 3.27E-07 | 96 |
| molecular function | GO:0008201 | heparin binding | 3.36E-07 | 49 |
| molecular function | GO:0015276 | ligand-gated ion channel activity | 4.55E-07 | 42 |
| molecular function | GO:0022834 | ligand-gated channel activity | 4.55E-07 | 42 |
| molecular function | GO:0008028 | monocarboxylic acid transmembrane transporter activity | 5.21E-07 | 22 |
| molecular function | GO:1901681 | sulfur compound binding | 5.38E-07 | 63 |
| molecular function | GO:0015103 | inorganic anion transmembrane transporter activity | 5.63E-07 | 46 |
| molecular function | GO:0031406 | carboxylic acid binding | 9.53E-07 | 51 |
| molecular function | GO:0022843 | voltage-gated cation channel activity | 1.02E-06 | 41 |
| molecular function | GO:0005253 | anion channel activity | 1.06E-06 | 31 |
| molecular function | GO:0015291 | secondary active transmembrane transporter activity | 1.35E-06 | 56 |
| molecular function | GO:0015079 | potassium ion transmembrane transporter activity | 1.82E-06 | 44 |
| molecular function | GO:0004867 | serine-type endopeptidase inhibitor activity | 2.15E-06 | 31 |
| molecular function | GO:0043177 | organic acid binding | 2.58E-06 | 52 |
| molecular function | GO:1901618 | organic hydroxy compound transmembrane transporter activity | 2.70E-06 | 19 |
| molecular function | GO:0005126 | cytokine receptor binding | 3.03E-06 | 72 |
