# Supplementary material for: TCGA based integrated genomic analyses of ceRNA network and novel subtypes revealing potential biomarkers for the prognosis and target therapy of tongue squamous cell carcinoma
Source: PLoS One. 2019 May 29;14(5):e0216834. doi: 10.1371/journal.pone.0216834 (PMC6541473; doi:10.1371/journal.pone.0216834)
Supplement: S2 Table — (DOCX) [file pone.0216834.s002.docx]

**S2 Table: KEGG pathways of DEmRNAs**

| ID | Description | P value | Count |
| --- | --- | --- | --- |
| hsa04974 | Protein digestion and absorption | 5.48E-13 | 41 |
| hsa04060 | Cytokine-cytokine receptor interaction | 4.84E-11 | 84 |
| hsa04512 | ECM-receptor interaction | 1.20E-09 | 34 |
| hsa04610 | Complement and coagulation cascades | 1.66E-06 | 28 |
| hsa05414 | Dilated cardiomyopathy (DCM) | 9.61E-06 | 29 |
| hsa05410 | Hypertrophic cardiomyopathy (HCM) | 1.59E-05 | 27 |
| hsa04510 | Focal adhesion | 2.44E-05 | 50 |
| hsa04970 | Salivary secretion | 2.84E-05 | 28 |
| hsa05322 | Systemic lupus erythematosus | 6.46E-05 | 36 |
| hsa00830 | Retinol metabolism | 8.19E-05 | 22 |
| hsa05412 | Arrhythmogenic right ventricular cardiomyopathy (ARVC) | 9.19E-05 | 23 |
| hsa04080 | Neuroactive ligand-receptor interaction | 0.000119 | 62 |
| hsa03320 | PPAR signaling pathway | 0.000147 | 23 |
| hsa04020 | Calcium signaling pathway | 0.000631 | 43 |
| hsa00350 | Tyrosine metabolism | 0.000848 | 13 |
| hsa00910 | Nitrogen metabolism | 0.00118 | 8 |
| hsa05323 | Rheumatoid arthritis | 0.001292 | 24 |
| hsa04261 | Adrenergic signaling in cardiomyocytes | 0.001538 | 34 |
| hsa04151 | PI3K-Akt signaling pathway | 0.001909 | 70 |
| hsa05033 | Nicotine addiction | 0.002557 | 13 |
| hsa04971 | Gastric acid secretion | 0.003171 | 20 |
| hsa04024 | cAMP signaling pathway | 0.004549 | 42 |
| hsa04657 | IL-17 signaling pathway | 0.004587 | 23 |
| hsa04260 | Cardiac muscle contraction | 0.005149 | 20 |
| hsa04727 | GABAergic synapse | 0.005529 | 22 |
| hsa00220 | Arginine biosynthesis | 0.00585 | 8 |
| hsa05202 | Transcriptional misregulation in cancer | 0.006847 | 39 |
| hsa00250 | Alanine, aspartate and glutamate metabolism | 0.007128 | 11 |
| hsa04976 | Bile secretion | 0.008747 | 18 |
| hsa04966 | Collecting duct acid secretion | 0.009565 | 9 |
| hsa00982 | Drug metabolism - cytochrome P450 | 0.01016 | 18 |
| hsa04964 | Proximal tubule bicarbonate reclamation | 0.010866 | 8 |
| hsa05031 | Amphetamine addiction | 0.01229 | 17 |
| hsa05146 | Amoebiasis | 0.013817 | 22 |
| hsa04514 | Cell adhesion molecules (CAMs) | 0.017811 | 30 |
| hsa05222 | Small cell lung cancer | 0.018699 | 21 |
| hsa05034 | Alcoholism | 0.018805 | 36 |
| hsa05150 | Staphylococcus aureus infection | 0.021909 | 14 |
| hsa05165 | Human papillomavirus infection | 0.022923 | 60 |
| hsa04950 | Maturity onset diabetes of the young | 0.023493 | 8 |
| hsa00512 | Mucin type O-glycan biosynthesis | 0.024384 | 9 |
| hsa04926 | Relaxin signaling pathway | 0.024606 | 27 |
| hsa04713 | Circadian entrainment | 0.026143 | 21 |
| hsa04972 | Pancreatic secretion | 0.026143 | 21 |
| hsa04380 | Osteoclast differentiation | 0.034827 | 26 |
| hsa00980 | Metabolism of xenobiotics by cytochrome P450 | 0.035035 | 17 |
| hsa04530 | Tight junction | 0.035115 | 33 |
| hsa05204 | Chemical carcinogenesis | 0.036419 | 18 |
| hsa00140 | Steroid hormone biosynthesis | 0.038218 | 14 |
| hsa04721 | Synaptic vesicle cycle | 0.043816 | 17 |
