# Supplementary material for: TCGA based integrated genomic analyses of ceRNA network and novel subtypes revealing potential biomarkers for the prognosis and target therapy of tongue squamous cell carcinoma
Source: PLoS One. 2019 May 29;14(5):e0216834. doi: 10.1371/journal.pone.0216834 (PMC6541473; doi:10.1371/journal.pone.0216834)
Supplement: S3 Table — (DOCX) [file pone.0216834.s003.docx]

**S3 Table: PPI network nodes of DEmRNAs**

| Symbol | Degree | Combined score | |
| --- | --- | --- | --- |
| ALB | 217 | 0.4 |  |
| FN1 | 174 | 0.41 |  |
| EGF | 147 | 0.4 |  |
| MMP9 | 122 | 0.4 |  |
| KNG1 | 115 | 0.413 |  |
| COL1A1 | 92 | 0.401 |  |
| SPP1 | 90 | 0.42 |  |
| ACTN2 | 86 | 0.58 |  |
| TTN | 86 | 0.503 |  |
| CXCL10 | 85 | 0.406 |  |
| F2 | 84 | 0.4 |  |
| AFP | 79 | 0.413 |  |
| IL17A | 79 | 0.41 |  |
| COL1A2 | 78 | 0.406 |  |
| COL3A1 | 78 | 0.402 |  |
| APOE | 78 | 0.4 |  |
| GNGT1 | 72 | 0.489 |  |
| ACTA1 | 72 | 0.415 |  |
| SPARC | 69 | 0.406 |  |
| CSF2 | 69 | 0.401 |  |
| ITGAX | 67 | 0.932 |  |
| SERPINE1 | 67 | 0.401 |  |
| ACAN | 66 | 0.455 |  |
| DMD | 66 | 0.414 |  |
| GNG7 | 66 | 0.411 |  |
| VCAN | 66 | 0.407 |  |
| PTGS2 | 65 | 0.413 |  |
| FPR2 | 65 | 0.4 |  |
| COL2A1 | 64 | 0.449 |  |
| MYL3 | 64 | 0.425 |  |
| MYH6 | 64 | 0.424 |  |
| PPBP | 64 | 0.413 |  |
| MYL1 | 64 | 0.412 |  |
| BGN | 64 | 0.405 |  |
| NGF | 64 | 0.4 |  |
| FGA | 63 | 0.43 |  |
| CAV3 | 62 | 0.402 |  |
| TAC1 | 61 | 0.433 |  |
| LDB3 | 61 | 0.432 |  |
| MYL2 | 61 | 0.401 |  |
| COL5A1 | 60 | 0.413 |  |
| DRD2 | 60 | 0.405 |  |
| GATA4 | 59 | 0.402 |  |
| IGFBP1 | 59 | 0.402 |  |
| POSTN | 59 | 0.401 |  |
| RYR1 | 59 | 0.401 |  |
| CFTR | 59 | 0.4 |  |
| TRDN | 58 | 0.539 |  |
| MYH7 | 58 | 0.41 |  |
| CXCL9 | 57 | 0.433 |  |
| ADIPOQ | 57 | 0.432 |  |
| MYOM2 | 57 | 0.413 |  |
| MMP3 | 57 | 0.404 |  |
| THY1 | 57 | 0.401 |  |
| CLU | 57 | 0.4 |  |
| PBX1 | 56 | 0.484 |  |
| ACTN3 | 56 | 0.456 |  |
| CP | 56 | 0.414 |  |
| MSLN | 55 | 0.513 |  |
| COL4A1 | 55 | 0.402 |  |
| APOA2 | 54 | 0.438 |  |
| COL6A1 | 54 | 0.419 |  |
| LUM | 54 | 0.409 |  |
| COL5A2 | 54 | 0.407 |  |
| COL4A2 | 54 | 0.4 |  |
| GRIA2 | 53 | 0.405 |  |
| TRIM63 | 52 | 0.457 |  |
| TCAP | 52 | 0.407 |  |
| NEB | 52 | 0.4 |  |
| TNNI2 | 51 | 0.436 |  |
| COL6A3 | 51 | 0.418 |  |
| MYBPC1 | 51 | 0.409 |  |
| TNNT3 | 51 | 0.409 |  |
| MYLPF | 50 | 0.407 |  |
| CHRM1 | 50 | 0.405 |  |
| MYH2 | 50 | 0.403 |  |
| THBS2 | 50 | 0.401 |  |
