# Supplementary material for: TCGA based integrated genomic analyses of ceRNA network and novel subtypes revealing potential biomarkers for the prognosis and target therapy of tongue squamous cell carcinoma
Source: PLoS One. 2019 May 29;14(5):e0216834. doi: 10.1371/journal.pone.0216834 (PMC6541473; doi:10.1371/journal.pone.0216834)
Supplement: S4 Table — (DOCX) [file pone.0216834.s004.docx]

**S4 Table: Survival analyses of DERNAs**

|  | Symbol | P value |
| --- | --- | --- |
| mRNA | NAGS | 0.000360865 |
|  | SOHLH1 | 0.000401198 |
|  | ETNPPL | 0.00046205 |
|  | CYP19A1 | 0.001100161 |
|  | TGM5 | 0.001104156 |
|  | UMODL1 | 0.001156322 |
|  | HIST1H3J | 0.002112268 |
|  | ODF4 | 0.002145965 |
|  | SLC16A1 | 0.002316955 |
|  | IRX4 | 0.002395528 |
|  | FSCN1 | 0.002554427 |
|  | SLC5A7 | 0.002575527 |
|  | CDCP2 | 0.002826027 |
|  | HIST1H2AG | 0.003533972 |
|  | E2F7 | 0.00361369 |
|  | SCN8A | 0.004154022 |
|  | HIST1H1D | 0.004304413 |
|  | AUNIP | 0.004468584 |
|  | NELL2 | 0.004520435 |
|  | ZIC2 | 0.004525664 |
|  | SPINK7 | 0.004824705 |
|  | CA9 | 0.005293387 |
|  | MAS1 | 0.005879025 |
|  | HIST1H3I | 0.006230366 |
|  | AC013470.2 | 0.006439851 |
|  | USP17L1 | 0.00692179 |
|  | PRELID3A | 0.0070006 |
|  | FUT6 | 0.00743154 |
|  | RNF224 | 0.008302427 |
|  | HOXC12 | 0.008787676 |
|  | SPC24 | 0.009530879 |
|  | KRT84 | 0.010047829 |
|  | CCL11 | 0.01005875 |
|  | KLF7 | 0.010638762 |
|  | CNFN | 0.010640736 |
|  | DMBX1 | 0.010824017 |
|  | C7orf33 | 0.011304774 |
|  | MEIS1 | 0.011455115 |
|  | MMP13 | 0.011539056 |
|  | ATOH7 | 0.011576168 |
|  | PFKM | 0.011963244 |
|  | ICAM5 | 0.012147153 |
|  | CD40LG | 0.012686374 |
|  | CHRM1 | 0.01321693 |
|  | CCNB2 | 0.013641314 |
|  | NR4A1 | 0.013801333 |
|  | FCN2 | 0.014002706 |
|  | KISS1R | 0.014816838 |
|  | BNC1 | 0.015087091 |
|  | BUB1 | 0.015754968 |
|  | ATP2B3 | 0.015964842 |
|  | SYNGR3 | 0.016087521 |
|  | PNCK | 0.016092106 |
|  | CLU | 0.01614936 |
|  | PRR11 | 0.016458732 |
|  | C14orf180 | 0.016908853 |
|  | PCP4 | 0.017260067 |
|  | KLHL14 | 0.017308538 |
|  | FAM25A | 0.01735488 |
|  | CKAP2L | 0.018244939 |
|  | HIST1H4I | 0.018491008 |
|  | CSAG2 | 0.018518861 |
|  | GFPT2 | 0.018612729 |
|  | ARHGEF39 | 0.018686755 |
|  | PKMYT1 | 0.018698772 |
|  | CIP2A | 0.019094722 |
|  | CEP55 | 0.019151239 |
|  | WNK4 | 0.019224809 |
|  | GRIN3B | 0.019389751 |
|  | PLK1 | 0.019454093 |
|  | VEGFD | 0.019708949 |
|  | HPN | 0.019961787 |
|  | SPRR2E | 0.020093855 |
|  | DKK1 | 0.020191983 |
|  | CLDN17 | 0.020322063 |
|  | PHYHIP | 0.022251282 |
|  | NDC80 | 0.023495945 |
|  | GNG8 | 0.023754471 |
|  | GTSF1L | 0.024255667 |
|  | S100A9 | 0.024516409 |
|  | GLOD5 | 0.024565816 |
|  | SLCO1B1 | 0.024883339 |
|  | ST6GALNAC1 | 0.025367504 |
|  | CGB5 | 0.025575786 |
|  | TIMP4 | 0.025776067 |
|  | IGF2BP3 | 0.025809809 |
|  | SLC2A14 | 0.026262725 |
|  | SYBU | 0.02638602 |
|  | L1CAM | 0.026575601 |
|  | STC2 | 0.026641136 |
|  | NRG3 | 0.028192764 |
|  | LEXM | 0.028836265 |
|  | CRCT1 | 0.028858706 |
|  | GOLGA6L2 | 0.029208484 |
|  | TMEM52 | 0.029817901 |
|  | TGM7 | 0.030069596 |
|  | CCL14 | 0.03009497 |
|  | MTHFD1L | 0.030716104 |
|  | HOXA1 | 0.031136569 |
|  | C14orf39 | 0.03114292 |
|  | DEPDC1B | 0.031420692 |
|  | LIPC | 0.031669866 |
|  | MAL | 0.033186621 |
|  | EXO1 | 0.033214602 |
|  | ABCA4 | 0.033388357 |
|  | CDC20 | 0.033549383 |
|  | KLF15 | 0.034454634 |
|  | ANGPTL3 | 0.034577336 |
|  | FHAD1 | 0.035158758 |
|  | PLIN4 | 0.035258464 |
|  | SP8 | 0.036022068 |
|  | MACROD1 | 0.036351961 |
|  | C11orf87 | 0.036605935 |
|  | HIST1H1B | 0.036637313 |
|  | SSX1 | 0.036739029 |
|  | MICB | 0.037197782 |
|  | PLAU | 0.03744232 |
|  | CCDC187 | 0.037448993 |
|  | EPHA8 | 0.037592608 |
|  | SPRR2A | 0.037724674 |
|  | COLGALT1 | 0.038432602 |
|  | KREMEN2 | 0.038443487 |
|  | KRTAP4-9 | 0.03863067 |
|  | IFNG | 0.038633582 |
|  | MPC1 | 0.038653101 |
|  | KCNJ16 | 0.038928365 |
|  | IFNL1 | 0.039209515 |
|  | FST | 0.039443286 |
|  | AQP7 | 0.041021934 |
|  | ADTRP | 0.042382142 |
|  | HOGA1 | 0.044478062 |
|  | LIFR | 0.044628471 |
|  | RDM1 | 0.044868613 |
|  | BIRC5 | 0.044917344 |
|  | OTOP2 | 0.045209912 |
|  | CCL17 | 0.046570353 |
|  | AC244517.10 | 0.046689229 |
|  | DPT | 0.046976868 |
|  | HIST1H2BK | 0.047021409 |
|  | HOXC4 | 0.047242186 |
|  | CCDC155 | 0.047564285 |
|  | FGA | 0.047625056 |
|  | MTFR2 | 0.048119876 |
|  | ZIM3 | 0.048382148 |
|  | FAM57B | 0.048440062 |
|  | IGF2BP2 | 0.048544646 |
|  | C9orf152 | 0.048737265 |
|  | SLC9A2 | 0.04901549 |
|  | ZNF98 | 0.049233983 |
|  | CENPA | 0.049465836 |
|  | ART4 | 0.049511607 |
|  | SPINK6 | 0.049538758 |
|  | TMEM89 | 0.049634119 |
|  | CD200R1L | 0.049905062 |
| miRNA | hsa-miR-337-3p | 0.007066876 |
|  | hsa-miR-654-3p | 0.009824125 |
|  | hsa-miR-1229-3p | 0.005321237 |
|  | hsa-miR-377-5p | 0.031090387 |
|  | hsa-miR-101-5p | 0.036480783 |
|  | hsa-miR-519a-5p | 0.049592266 |
| lncRNA | AL359851.1 | 0.000961755 |
|  | LINC02560 | 0.001813109 |
|  | AC009226.1 | 0.004008458 |
|  | AL135999.3 | 0.00450135 |
|  | FOXD2-AS1 | 0.005301862 |
|  | AL158209.1 | 0.00575772 |
|  | AC099850.3 | 0.006559458 |
|  | AC108474.1 | 0.008500586 |
|  | AC156455.1 | 0.008856256 |
|  | MIR4713HG | 0.009382198 |
|  | AC012456.2 | 0.009910569 |
|  | LINC01555 | 0.012196752 |
|  | LINC02477 | 0.013602471 |
|  | LHFPL3-AS1 | 0.014190839 |
|  | HOXA10-AS | 0.015607554 |
|  | LINC00659 | 0.015683572 |
|  | AC022031.2 | 0.016116974 |
|  | LINC02028 | 0.016551484 |
|  | RNU6ATAC35P | 0.018228842 |
|  | AC084880.4 | 0.018409061 |
|  | AP005131.2 | 0.018505279 |
|  | FGF12-AS2 | 0.020071807 |
|  | AL391807.1 | 0.020079568 |
|  | FLJ42969 | 0.020993334 |
|  | AC044784.1 | 0.021148186 |
|  | AL138916.1 | 0.022586798 |
|  | AP001790.1 | 0.02283763 |
|  | AC009264.1 | 0.023342443 |
|  | AC008147.2 | 0.024166573 |
|  | PART1 | 0.024303417 |
|  | AL121832.3 | 0.024751412 |
|  | AC009560.1 | 0.024960318 |
|  | LINC00636 | 0.024979642 |
|  | AC019131.1 | 0.025970536 |
|  | AP005209.1 | 0.026047016 |
|  | KCNQ5-IT1 | 0.026198408 |
|  | AL391001.1 | 0.027242877 |
|  | LINC01615 | 0.028488602 |
|  | LINC00443 | 0.030304012 |
|  | LINC02461 | 0.031459278 |
|  | AC090337.1 | 0.031554789 |
|  | AP000820.1 | 0.032355245 |
|  | LINC02487 | 0.032572701 |
|  | AL139352.1 | 0.033335087 |
|  | SLC8A1-AS1 | 0.033813637 |
|  | LINC01983 | 0.033839691 |
|  | EGFR-AS1 | 0.034976365 |
|  | AC020634.1 | 0.036496157 |
|  | LINC00958 | 0.036538709 |
|  | AC020913.3 | 0.036958822 |
|  | DLEU7-AS1 | 0.037065275 |
|  | AC009093.6 | 0.037079614 |
|  | AC138904.1 | 0.038303696 |
|  | AC092070.4 | 0.038622178 |
|  | AC108861.1 | 0.039202195 |
|  | AL513318.2 | 0.039347824 |
|  | AC011611.3 | 0.039351719 |
|  | AC017048.3 | 0.039608427 |
|  | AC007336.1 | 0.040242907 |
|  | AC087190.2 | 0.042349247 |
|  | AC118754.1 | 0.042969083 |
|  | LINC00908 | 0.043044021 |
|  | HOXD-AS2 | 0.043544208 |
|  | AC108865.1 | 0.044079905 |
|  | AC012213.4 | 0.044311705 |
|  | AC048341.2 | 0.045063847 |
|  | AC131157.1 | 0.045302247 |
|  | AL365356.3 | 0.045719881 |
|  | AL049536.1 | 0.046004102 |
|  | AC034223.2 | 0.046181831 |
|  | AC015845.2 | 0.046617399 |
|  | AL022326.2 | 0.046772333 |
|  | AC020907.4 | 0.047670089 |
|  | AC139749.1 | 0.04787012 |
|  | AL356270.1 | 0.047953956 |
|  | NAALADL2-AS2 | 0.049788173 |
