# Supplementary material for: TCGA based integrated genomic analyses of ceRNA network and novel subtypes revealing potential biomarkers for the prognosis and target therapy of tongue squamous cell carcinoma
Source: PLoS One. 2019 May 29;14(5):e0216834. doi: 10.1371/journal.pone.0216834 (PMC6541473; doi:10.1371/journal.pone.0216834)
Supplement: S5 Table — (DOCX) [file pone.0216834.s005.docx]

**S5 Table: 358 pairs of DEmiRNA-DEmRNA interactions**

| miRNA | mRNA |
| --- | --- |
| hsa-let-7c-5p | ACTA1 |
| hsa-let-7c-5p | AURKA |
| hsa-let-7c-5p | BASP1 |
| hsa-let-7c-5p | CEP55 |
| hsa-let-7c-5p | CFL2 |
| hsa-let-7c-5p | CPEB3 |
| hsa-let-7c-5p | DLG2 |
| hsa-let-7c-5p | E2F7 |
| hsa-let-7c-5p | EFHC2 |
| hsa-let-7c-5p | FYCO1 |
| hsa-let-7c-5p | GATM |
| hsa-let-7c-5p | GJC1 |
| hsa-let-7c-5p | HOXA5 |
| hsa-let-7c-5p | IFI44L |
| hsa-let-7c-5p | IQGAP2 |
| hsa-let-7c-5p | KCNJ11 |
| hsa-let-7c-5p | NR4A1 |
| hsa-let-7c-5p | ONECUT2 |
| hsa-let-7c-5p | PTPRD |
| hsa-let-7c-5p | SLC39A14 |
| hsa-let-7c-5p | SOX11 |
| hsa-let-7c-5p | SVIL |
| hsa-let-7c-5p | TGFBR3 |
| hsa-let-7c-5p | TNFSF9 |
| hsa-let-7c-5p | TSPAN12 |
| hsa-miR-101-3p | CCNE1 |
| hsa-miR-101-3p | CEP55 |
| hsa-miR-101-3p | CPEB3 |
| hsa-miR-101-3p | DCBLD2 |
| hsa-miR-101-3p | DEPDC1B |
| hsa-miR-101-3p | DUSP1 |
| hsa-miR-101-3p | EPDR1 |
| hsa-miR-101-3p | EYA1 |
| hsa-miR-101-3p | FHL1 |
| hsa-miR-101-3p | FOS |
| hsa-miR-101-3p | FOXD1 |
| hsa-miR-101-3p | GJA1 |
| hsa-miR-101-3p | HMGA2 |
| hsa-miR-101-3p | HOXA10 |
| hsa-miR-101-3p | HOXA9 |
| hsa-miR-101-3p | ID4 |
| hsa-miR-101-3p | IKZF2 |
| hsa-miR-101-3p | LIFR |
| hsa-miR-101-3p | MAML3 |
| hsa-miR-101-3p | MNX1 |
| hsa-miR-101-3p | NETO2 |
| hsa-miR-101-3p | ONECUT2 |
| hsa-miR-101-3p | PCDH7 |
| hsa-miR-101-3p | PKIA |
| hsa-miR-101-3p | PLK1 |
| hsa-miR-101-3p | SALL1 |
| hsa-miR-101-3p | SLAIN1 |
| hsa-miR-101-3p | SLC1A1 |
| hsa-miR-101-3p | STC2 |
| hsa-miR-101-3p | TGFBR3 |
| hsa-miR-101-3p | TSPAN12 |
| hsa-miR-101-3p | VCAN |
| hsa-miR-101-3p | ZIC5 |
| hsa-miR-127-3p | DUSP1 |
| hsa-miR-127-3p | HOXB7 |
| hsa-miR-135a-5p | ADA |
| hsa-miR-135a-5p | ANLN |
| hsa-miR-135a-5p | BIRC5 |
| hsa-miR-135a-5p | CDC6 |
| hsa-miR-135a-5p | CERCAM |
| hsa-miR-135a-5p | DCBLD2 |
| hsa-miR-135a-5p | HOXA10 |
| hsa-miR-135a-5p | IGF2BP1 |
| hsa-miR-135a-5p | NETO2 |
| hsa-miR-135a-5p | NR4A2 |
| hsa-miR-135a-5p | OAS3 |
| hsa-miR-135a-5p | SHROOM3 |
| hsa-miR-135a-5p | SLC35F1 |
| hsa-miR-135a-5p | TNC |
| hsa-miR-135a-5p | TRIP13 |
| hsa-miR-136-5p | C12orf75 |
| hsa-miR-136-5p | CDH2 |
| hsa-miR-136-5p | DUSP1 |
| hsa-miR-136-5p | KRT10 |
| hsa-miR-136-5p | MTBP |
| hsa-miR-136-5p | RNF157 |
| hsa-miR-136-5p | SLC16A9 |
| hsa-miR-136-5p | VCAN |
| hsa-miR-18a-5p | CA2 |
| hsa-miR-18a-5p | DCBLD2 |
| hsa-miR-18a-5p | E2F7 |
| hsa-miR-18a-5p | ENPP5 |
| hsa-miR-18a-5p | F2R |
| hsa-miR-18a-5p | FBN1 |
| hsa-miR-18a-5p | FYCO1 |
| hsa-miR-18a-5p | HIST1H4H |
| hsa-miR-18a-5p | HOXA9 |
| hsa-miR-18a-5p | KIF2C |
| hsa-miR-18a-5p | KIT |
| hsa-miR-18a-5p | LPIN1 |
| hsa-miR-18a-5p | PPP1R12B |
| hsa-miR-18a-5p | RAD51AP1 |
| hsa-miR-18a-5p | RNF157 |
| hsa-miR-18a-5p | SH3BGR |
| hsa-miR-18a-5p | SYNM |
| hsa-miR-18a-5p | TGFBR3 |
| hsa-miR-18a-5p | TSHZ3 |
| hsa-miR-208a-3p | AMOT |
| hsa-miR-208a-3p | CFL2 |
| hsa-miR-208a-3p | DEPDC1B |
| hsa-miR-208a-3p | EYA4 |
| hsa-miR-208a-3p | HIST1H3B |
| hsa-miR-208a-3p | KIF23 |
| hsa-miR-208a-3p | KIF2C |
| hsa-miR-208a-3p | SALL1 |
| hsa-miR-208a-3p | SYBU |
| hsa-miR-208a-3p | TSPAN12 |
| hsa-miR-208b-3p | DEPDC1B |
| hsa-miR-208b-3p | EYA4 |
| hsa-miR-208b-3p | HIST1H3B |
| hsa-miR-208b-3p | KIF23 |
| hsa-miR-208b-3p | SALL1 |
| hsa-miR-208b-3p | SYBU |
| hsa-miR-208b-3p | TSPAN12 |
| hsa-miR-210-3p | BAMBI |
| hsa-miR-210-3p | DCBLD1 |
| hsa-miR-210-3p | DLX1 |
| hsa-miR-210-3p | ITGA6 |
| hsa-miR-210-3p | SHCBP1 |
| hsa-miR-224-5p | AGRN |
| hsa-miR-224-5p | CA2 |
| hsa-miR-224-5p | DCBLD2 |
| hsa-miR-224-5p | ELAVL2 |
| hsa-miR-224-5p | EYA4 |
| hsa-miR-224-5p | GPR176 |
| hsa-miR-224-5p | HOXA5 |
| hsa-miR-224-5p | HOXA9 |
| hsa-miR-224-5p | IGF2BP1 |
| hsa-miR-224-5p | KIF23 |
| hsa-miR-224-5p | MELK |
| hsa-miR-224-5p | NETO2 |
| hsa-miR-224-5p | RRAGD |
| hsa-miR-29c-3p | ADA |
| hsa-miR-29c-3p | AMOT |
| hsa-miR-29c-3p | CD276 |
| hsa-miR-29c-3p | COL4A1 |
| hsa-miR-29c-3p | COL4A2 |
| hsa-miR-29c-3p | CPEB3 |
| hsa-miR-29c-3p | E2F7 |
| hsa-miR-29c-3p | HOXA10 |
| hsa-miR-29c-3p | KIF23 |
| hsa-miR-29c-3p | MICB |
| hsa-miR-29c-3p | MYBL2 |
| hsa-miR-29c-3p | NREP |
| hsa-miR-29c-3p | SALL1 |
| hsa-miR-29c-3p | SERPINH1 |
| hsa-miR-29c-3p | SLC16A1 |
| hsa-miR-29c-3p | SLC16A14 |
| hsa-miR-29c-3p | SPARC |
| hsa-miR-29c-3p | SPRY4 |
| hsa-miR-29c-3p | TSPAN12 |
| hsa-miR-29c-3p | ULBP2 |
| hsa-miR-29c-3p | ZFP36 |
| hsa-miR-29c-3p | ZIC5 |
| hsa-miR-30e-5p | CDH2 |
| hsa-miR-30e-5p | CELSR3 |
| hsa-miR-30e-5p | CFL2 |
| hsa-miR-30e-5p | CHPT1 |
| hsa-miR-30e-5p | DCBLD1 |
| hsa-miR-30e-5p | DCBLD2 |
| hsa-miR-30e-5p | DDAH1 |
| hsa-miR-30e-5p | DLX1 |
| hsa-miR-30e-5p | DLX2 |
| hsa-miR-30e-5p | E2F7 |
| hsa-miR-30e-5p | ENPP4 |
| hsa-miR-30e-5p | FOXA1 |
| hsa-miR-30e-5p | FOXC1 |
| hsa-miR-30e-5p | FYCO1 |
| hsa-miR-30e-5p | GFPT2 |
| hsa-miR-30e-5p | HMGA2 |
| hsa-miR-30e-5p | ID4 |
| hsa-miR-30e-5p | IKBIP |
| hsa-miR-30e-5p | LIFR |
| hsa-miR-30e-5p | LIN28B |
| hsa-miR-30e-5p | LPCAT1 |
| hsa-miR-30e-5p | LRRC8C |
| hsa-miR-30e-5p | MICB |
| hsa-miR-30e-5p | MYBL2 |
| hsa-miR-30e-5p | NID1 |
| hsa-miR-30e-5p | NR4A2 |
| hsa-miR-30e-5p | PITX2 |
| hsa-miR-30e-5p | PLEKHA7 |
| hsa-miR-30e-5p | PRDM13 |
| hsa-miR-30e-5p | RHEBL1 |
| hsa-miR-30e-5p | RNF157 |
| hsa-miR-30e-5p | RRAGD |
| hsa-miR-30e-5p | SLAIN1 |
| hsa-miR-30e-5p | SNX10 |
| hsa-miR-30e-5p | SOBP |
| hsa-miR-30e-5p | SOCS1 |
| hsa-miR-30e-5p | TRIM71 |
| hsa-miR-30e-5p | ZIC2 |
| hsa-miR-337-3p | AMOT |
| hsa-miR-34b-5p | CDH2 |
| hsa-miR-34b-5p | CEP55 |
| hsa-miR-34b-5p | SYBU |
| hsa-miR-378c | CPEB3 |
| hsa-miR-378f | CPEB3 |
| hsa-miR-379-5p | GATM |
| hsa-miR-424-5p | AMOT |
| hsa-miR-424-5p | ANLN |
| hsa-miR-424-5p | CA2 |
| hsa-miR-424-5p | CA8 |
| hsa-miR-424-5p | CACNA2D3 |
| hsa-miR-424-5p | CCNE1 |
| hsa-miR-424-5p | CEP55 |
| hsa-miR-424-5p | CHPT1 |
| hsa-miR-424-5p | CLSPN |
| hsa-miR-424-5p | CLU |
| hsa-miR-424-5p | CPEB3 |
| hsa-miR-424-5p | DCBLD2 |
| hsa-miR-424-5p | DEPDC1B |
| hsa-miR-424-5p | DYRK1B |
| hsa-miR-424-5p | E2F7 |
| hsa-miR-424-5p | EFCAB6 |
| hsa-miR-424-5p | EIF5A2 |
| hsa-miR-424-5p | EN2 |
| hsa-miR-424-5p | ENPP4 |
| hsa-miR-424-5p | EYA1 |
| hsa-miR-424-5p | FOXC1 |
| hsa-miR-424-5p | GNA12 |
| hsa-miR-424-5p | HMGA2 |
| hsa-miR-424-5p | HOXA10 |
| hsa-miR-424-5p | HOXC8 |
| hsa-miR-424-5p | HOXD13 |
| hsa-miR-424-5p | KIF18B |
| hsa-miR-424-5p | KIF23 |
| hsa-miR-424-5p | KIF5C |
| hsa-miR-424-5p | LY6E |
| hsa-miR-424-5p | MICB |
| hsa-miR-424-5p | MOB3B |
| hsa-miR-424-5p | MYB |
| hsa-miR-424-5p | MYO5B |
| hsa-miR-424-5p | NAT8L |
| hsa-miR-424-5p | NR4A1 |
| hsa-miR-424-5p | ONECUT2 |
| hsa-miR-424-5p | PAX9 |
| hsa-miR-424-5p | PDK4 |
| hsa-miR-424-5p | PIP5K1B |
| hsa-miR-424-5p | PLS1 |
| hsa-miR-424-5p | PMEPA1 |
| hsa-miR-424-5p | PTK7 |
| hsa-miR-424-5p | RAB9B |
| hsa-miR-424-5p | SALL1 |
| hsa-miR-424-5p | SCN8A |
| hsa-miR-424-5p | SH3BGRL2 |
| hsa-miR-424-5p | SLC7A2 |
| hsa-miR-424-5p | SPARC |
| hsa-miR-424-5p | SPRY4 |
| hsa-miR-424-5p | STK33 |
| hsa-miR-424-5p | TFPI2 |
| hsa-miR-424-5p | TGFBR3 |
| hsa-miR-424-5p | TMEM100 |
| hsa-miR-424-5p | TNFSF9 |
| hsa-miR-424-5p | VCAN |
| hsa-miR-433-3p | CA2 |
| hsa-miR-433-3p | E2F7 |
| hsa-miR-433-3p | EYA4 |
| hsa-miR-433-3p | HOXA10 |
| hsa-miR-433-3p | HOXA5 |
| hsa-miR-433-3p | SNX10 |
| hsa-miR-450a-5p | TRIP13 |
| hsa-miR-488-3p | CSTB |
| hsa-miR-488-3p | DDAH1 |
| hsa-miR-488-3p | HOXB8 |
| hsa-miR-488-3p | KIF23 |
| hsa-miR-488-3p | NREP |
| hsa-miR-488-3p | OCLN |
| hsa-miR-488-3p | RAB11FIP1 |
| hsa-miR-488-3p | SLAIN1 |
| hsa-miR-488-3p | STC2 |
| hsa-miR-488-3p | SYDE2 |
| hsa-miR-488-3p | TSPAN12 |
| hsa-miR-488-3p | ZIC5 |
| hsa-miR-493-5p | CDKN3 |
| hsa-miR-499a-5p | BAMBI |
| hsa-miR-499a-5p | BIRC5 |
| hsa-miR-499a-5p | CFL2 |
| hsa-miR-499a-5p | DEPDC1B |
| hsa-miR-499a-5p | EYA4 |
| hsa-miR-499a-5p | FOS |
| hsa-miR-499a-5p | HIST1H3B |
| hsa-miR-499a-5p | HOXA10 |
| hsa-miR-499a-5p | IKBIP |
| hsa-miR-499a-5p | IKZF2 |
| hsa-miR-499a-5p | KIF23 |
| hsa-miR-499a-5p | MELK |
| hsa-miR-499a-5p | SALL1 |
| hsa-miR-499a-5p | SLC16A6 |
| hsa-miR-499a-5p | SOX11 |
| hsa-miR-499a-5p | SYBU |
| hsa-miR-503-5p | ANLN |
| hsa-miR-503-5p | CA2 |
| hsa-miR-503-5p | CCNE1 |
| hsa-miR-503-5p | CLEC4F |
| hsa-miR-503-5p | CPEB3 |
| hsa-miR-503-5p | DUSP5 |
| hsa-miR-503-5p | DYRK1B |
| hsa-miR-503-5p | E2F7 |
| hsa-miR-503-5p | HMGA2 |
| hsa-miR-503-5p | HOXA10 |
| hsa-miR-503-5p | HOXC8 |
| hsa-miR-503-5p | KIF23 |
| hsa-miR-503-5p | KIF5C |
| hsa-miR-503-5p | MOB3B |
| hsa-miR-503-5p | MYB |
| hsa-miR-503-5p | PIP5K1B |
| hsa-miR-503-5p | PTK7 |
| hsa-miR-503-5p | RAB9B |
| hsa-miR-503-5p | RRAGD |
| hsa-miR-503-5p | SALL1 |
| hsa-miR-503-5p | SCN8A |
| hsa-miR-503-5p | SPARC |
| hsa-miR-503-5p | STK33 |
| hsa-miR-503-5p | TFPI2 |
| hsa-miR-503-5p | TMEM100 |
| hsa-miR-512-3p | CDCA8 |
| hsa-miR-512-3p | HOXD8 |
| hsa-miR-512-3p | MICAL2 |
| hsa-miR-512-3p | PPP1R12B |
| hsa-miR-512-3p | TNFRSF12A |
| hsa-miR-520c-3p | AGFG2 |
| hsa-miR-520c-3p | ASB14 |
| hsa-miR-520c-3p | BAMBI |
| hsa-miR-520c-3p | CEP55 |
| hsa-miR-520c-3p | CFL2 |
| hsa-miR-520c-3p | DUSP1 |
| hsa-miR-520c-3p | E2F7 |
| hsa-miR-520c-3p | ENPP5 |
| hsa-miR-520c-3p | FYCO1 |
| hsa-miR-520c-3p | HOXD8 |
| hsa-miR-520c-3p | IGF2BP1 |
| hsa-miR-520c-3p | KAT2B |
| hsa-miR-520c-3p | KIF23 |
| hsa-miR-520c-3p | MICB |
| hsa-miR-520c-3p | NETO2 |
| hsa-miR-520c-3p | PKIA |
| hsa-miR-520c-3p | PPM1L |
| hsa-miR-520c-3p | PPP1R9A |
| hsa-miR-520c-3p | RAB11FIP1 |
| hsa-miR-520c-3p | RRAGD |
| hsa-miR-520c-3p | SERPINH1 |
| hsa-miR-520c-3p | SHCBP1 |
| hsa-miR-520c-3p | SLAIN1 |
| hsa-miR-520c-3p | SLC16A9 |
| hsa-miR-520c-3p | SYDE2 |
| hsa-miR-520c-3p | TTC9 |
| hsa-miR-520f-3p | ASB14 |
| hsa-miR-520f-3p | CDCA8 |
| hsa-miR-520f-3p | FYCO1 |
| hsa-miR-520f-3p | HOXD8 |
| hsa-miR-520f-3p | TTC9 |
| hsa-miR-99a-5p | EPDR1 |
