# Supplementary material for: TCGA based integrated genomic analyses of ceRNA network and novel subtypes revealing potential biomarkers for the prognosis and target therapy of tongue squamous cell carcinoma
Source: PLoS One. 2019 May 29;14(5):e0216834. doi: 10.1371/journal.pone.0216834 (PMC6541473; doi:10.1371/journal.pone.0216834)
Supplement: S6 Table — (DOCX) [file pone.0216834.s006.docx]

**S6 Table:** **44 pairs of** **DEmiRNA-DElncRNA interactions**

| miRNA | lncRNA |
| --- | --- |
| hsa-miR-424-5p | AC003092.1 |
| hsa-miR-195-5p | AC003092.1 |
| hsa-miR-520c-3p | AC005083.1 |
| hsa-miR-299-3p | AC005083.1 |
| hsa-miR-31-5p | AC017048.3 |
| hsa-miR-18a-5p | AC156455.1 |
| hsa-miR-7-5p | AP001619.2 |
| hsa-miR-424-5p | C1RL-AS1 |
| hsa-miR-195-5p | C1RL-AS1 |
| hsa-let-7c-5p | CDKN2B-AS1 |
| hsa-miR-424-5p | DLX6-AS1 |
| hsa-miR-195-5p | DLX6-AS1 |
| hsa-miR-301a-3p | HOTAIR |
| hsa-miR-148a-3p | HOTAIR |
| hsa-miR-148a-3p | HOTAIRM1 |
| hsa-miR-615-3p | HOTTIP |
| hsa-miR-301a-3p | HOXA11-AS |
| hsa-miR-208b-3p | HOXA11-AS |
| hsa-miR-208a-3p | HOXA11-AS |
| hsa-miR-148a-3p | HOXA11-AS |
| hsa-let-7c-5p | HOXA11-AS |
| hsa-miR-29c-3p | HOXA-AS3 |
| hsa-miR-29c-3p | LIFR-AS1 |
| hsa-miR-31-5p | LINC00174 |
| hsa-miR-148a-3p | LINC00174 |
| hsa-miR-411-5p | LINC00461 |
| hsa-miR-520c-3p | LINC00472 |
| hsa-miR-381-3p | LINC00472 |
| hsa-miR-204-5p | LINC00472 |
| hsa-miR-196b-5p | LINC00472 |
| hsa-miR-196a-5p | LINC00472 |
| hsa-miR-18a-5p | LINC00622 |
| hsa-miR-539-5p | LINC00707 |
| hsa-miR-424-5p | LINC00707 |
| hsa-miR-376c-3p | LINC00707 |
| hsa-miR-30e-5p | LINC00707 |
| hsa-miR-30a-5p | LINC00707 |
| hsa-miR-195-5p | LINC00707 |
| hsa-miR-301a-3p | LINC00839 |
| hsa-miR-424-5p | MEG8 |
| hsa-miR-195-5p | MEG8 |
| hsa-miR-30e-5p | SLC26A4-AS1 |
| hsa-miR-30a-5p | SLC26A4-AS1 |
| hsa-miR-301a-3p | SLC26A4-AS1 |
