# Supplementary material for: TCGA based integrated genomic analyses of ceRNA network and novel subtypes revealing potential biomarkers for the prognosis and target therapy of tongue squamous cell carcinoma
Source: PLoS One. 2019 May 29;14(5):e0216834. doi: 10.1371/journal.pone.0216834 (PMC6541473; doi:10.1371/journal.pone.0216834)
Supplement: S7 Table — (DOCX) [file pone.0216834.s007.docx]

**S7 Table: Functional enrichment analyses of GO and KEGG in subtype A**

| Type | ID | Description | P value | Count |
| --- | --- | --- | --- | --- |
| biological process | GO:0007586 | digestion | 1.09E-08 | 16 |
| biological process | GO:0006805 | xenobiotic metabolic process | 8.04E-08 | 14 |
| biological process | GO:0022600 | digestive system process | 4.75E-07 | 12 |
| biological process | GO:0016266 | O-glycan processing | 2.95E-06 | 9 |
| biological process | GO:0019748 | secondary metabolic process | 3.40E-06 | 9 |
| biological process | GO:0048665 | neuron fate specification | 3.81E-06 | 7 |
| biological process | GO:0048663 | neuron fate commitment | 6.56E-06 | 9 |
| biological process | GO:0071466 | cellular response to xenobiotic stimulus | 8.10E-06 | 14 |
| biological process | GO:0048871 | multicellular organismal homeostasis | 2.48E-05 | 24 |
| biological process | GO:0052695 | cellular glucuronidation | 2.73E-05 | 5 |
| biological process | GO:0042445 | hormone metabolic process | 3.09E-05 | 15 |
| biological process | GO:0030277 | maintenance of gastrointestinal epithelium | 3.59E-05 | 5 |
| biological process | GO:0001894 | tissue homeostasis | 3.63E-05 | 15 |
| biological process | GO:0052697 | xenobiotic glucuronidation | 4.46E-05 | 4 |
| cellular component | GO:0016327 | apicolateral plasma membrane | 2.15E-05 | 5 |
| cellular component | GO:0098793 | presynapse | 4.28E-05 | 24 |
| cellular component | GO:0099699 | integral component of synaptic membrane | 6.44E-05 | 12 |
| molecular function | GO:0020037 | heme binding | 1.61E-05 | 12 |
| molecular function | GO:0022839 | ion gated channel activity | 2.76E-05 | 20 |
| molecular function | GO:0001972 | retinoic acid binding | 3.21E-05 | 5 |
| molecular function | GO:0046906 | tetrapyrrole binding | 3.37E-05 | 12 |
| molecular function | GO:0015108 | chloride transmembrane transporter activity | 3.86E-05 | 10 |
| molecular function | GO:0022836 | gated channel activity | 3.98E-05 | 20 |
| molecular function | GO:0019825 | oxygen binding | 8.19E-05 | 6 |
| molecular function | GO:0015103 | inorganic anion transmembrane transporter activity | 9.62E-05 | 12 |
| KEGG pathway | hsa05204 | Chemical carcinogenesis | 5.84E-08 | 12 |
| KEGG pathway | hsa00980 | Metabolism of xenobiotics by cytochrome P450 | 2.40E-07 | 11 |
| KEGG pathway | hsa00830 | Retinol metabolism | 6.44E-07 | 10 |
| KEGG pathway | hsa05033 | Nicotine addiction | 1.11E-05 | 7 |
| KEGG pathway | hsa00053 | Ascorbate and aldarate metabolism | 1.14E-05 | 6 |
| KEGG pathway | hsa00982 | Drug metabolism - cytochrome P450 | 8.00E-05 | 8 |
| KEGG pathway | hsa04950 | Maturity onset diabetes of the young | 0.000134 | 5 |
| KEGG pathway | hsa00983 | Drug metabolism - other enzymes | 0.000155 | 8 |
| KEGG pathway | hsa00140 | Steroid hormone biosynthesis | 0.000166 | 7 |
| KEGG pathway | hsa04080 | Neuroactive ligand-receptor interaction | 0.000349 | 17 |
| KEGG pathway | hsa00040 | Pentose and glucuronate interconversions | 0.000497 | 5 |
| KEGG pathway | hsa00860 | Porphyrin and chlorophyll metabolism | 0.001339 | 5 |
| KEGG pathway | hsa04970 | Salivary secretion | 0.001958 | 7 |
| KEGG pathway | hsa05030 | Cocaine addiction | 0.002687 | 5 |
