# Supplementary material for: TCGA based integrated genomic analyses of ceRNA network and novel subtypes revealing potential biomarkers for the prognosis and target therapy of tongue squamous cell carcinoma
Source: PLoS One. 2019 May 29;14(5):e0216834. doi: 10.1371/journal.pone.0216834 (PMC6541473; doi:10.1371/journal.pone.0216834)
Supplement: S8 Table — (DOCX) [file pone.0216834.s008.docx]

**S8 Table: Functional enrichment analyses of GO and KEGG in subtype B**

| Type | ID | Description | P value | Count |
| --- | --- | --- | --- | --- |
| biological process | GO:0030216 | keratinocyte differentiation | 1.12E-81 | 73 |
| biological process | GO:0031424 | keratinization | 1.72E-79 | 66 |
| biological process | GO:0009913 | epidermal cell differentiation | 2.77E-79 | 75 |
| biological process | GO:0043588 | skin development | 1.00E-76 | 77 |
| biological process | GO:0008544 | epidermis development | 5.80E-73 | 77 |
| biological process | GO:0018149 | peptide cross-linking | 1.56E-61 | 38 |
| biological process | GO:0070268 | cornification | 3.04E-53 | 41 |
| biological process | GO:0019730 | antimicrobial humoral response | 2.45E-11 | 14 |
| biological process | GO:0031640 | killing of cells of other organism | 7.25E-10 | 10 |
| biological process | GO:0044364 | disruption of cells of other organism | 7.25E-10 | 10 |
| biological process | GO:0042742 | defense response to bacterium | 1.18E-09 | 19 |
| biological process | GO:0006959 | humoral immune response | 5.18E-09 | 19 |
| biological process | GO:0035821 | modification of morphology or physiology of other organism | 1.53E-08 | 13 |
| biological process | GO:0097530 | granulocyte migration | 1.74E-08 | 12 |
| biological process | GO:0061436 | establishment of skin barrier | 4.23E-08 | 6 |
| biological process | GO:0071621 | granulocyte chemotaxis | 5.47E-08 | 11 |
| biological process | GO:0033561 | regulation of water loss via skin | 8.00E-08 | 6 |
| biological process | GO:0050832 | defense response to fungus | 1.45E-07 | 7 |
| biological process | GO:1990266 | neutrophil migration | 2.87E-07 | 10 |
| biological process | GO:0060326 | cell chemotaxis | 5.44E-07 | 15 |
| biological process | GO:0050830 | defense response to Gram-positive bacterium | 6.30E-07 | 9 |
| biological process | GO:0061844 | antimicrobial humoral immune response mediated by antimicrobial peptide | 7.68E-07 | 8 |
| biological process | GO:0001906 | cell killing | 8.97E-07 | 11 |
| biological process | GO:0009620 | response to fungus | 9.83E-07 | 7 |
| biological process | GO:0030593 | neutrophil chemotaxis | 1.16E-06 | 9 |
| biological process | GO:0097529 | myeloid leukocyte migration | 1.31E-06 | 12 |
| biological process | GO:0010951 | negative regulation of endopeptidase activity | 2.48E-06 | 13 |
| biological process | GO:0030595 | leukocyte chemotaxis | 3.24E-06 | 12 |
| biological process | GO:0050829 | defense response to Gram-negative bacterium | 3.65E-06 | 8 |
| biological process | GO:0010466 | negative regulation of peptidase activity | 3.98E-06 | 13 |
| biological process | GO:0050891 | multicellular organismal water homeostasis | 4.22E-06 | 7 |
| biological process | GO:0030104 | water homeostasis | 6.41E-06 | 7 |
| biological process | GO:0036152 | phosphatidylethanolamine acyl-chain remodeling | 6.64E-06 | 5 |
| biological process | GO:0036151 | phosphatidylcholine acyl-chain remodeling | 8.07E-06 | 5 |
| biological process | GO:0036149 | phosphatidylinositol acyl-chain remodeling | 2.51E-05 | 4 |
| biological process | GO:0045861 | negative regulation of proteolysis | 2.72E-05 | 14 |
| biological process | GO:0036148 | phosphatidylglycerol acyl-chain remodeling | 3.20E-05 | 4 |
| biological process | GO:0052548 | regulation of endopeptidase activity | 3.57E-05 | 15 |
| biological process | GO:0036150 | phosphatidylserine acyl-chain remodeling | 6.11E-05 | 4 |
| biological process | GO:0052547 | regulation of peptidase activity | 6.90E-05 | 15 |
| biological process | GO:0051346 | negative regulation of hydrolase activity | 0.000103 | 15 |
| biological process | GO:0016042 | lipid catabolic process | 0.000153 | 12 |
| biological process | GO:0045104 | intermediate filament cytoskeleton organization | 0.000158 | 5 |
| biological process | GO:0050900 | leukocyte migration | 0.000164 | 15 |
| biological process | GO:0045103 | intermediate filament-based process | 0.000174 | 5 |
| biological process | GO:0046470 | phosphatidylcholine metabolic process | 0.000182 | 6 |
| biological process | GO:0045682 | regulation of epidermis development | 0.000208 | 6 |
| biological process | GO:0046471 | phosphatidylglycerol metabolic process | 0.000227 | 4 |
| biological process | GO:0006658 | phosphatidylserine metabolic process | 0.00026 | 4 |
| biological process | GO:0050482 | arachidonic acid secretion | 0.00026 | 4 |
| biological process | GO:1903963 | arachidonate transport | 0.00026 | 4 |
| cellular component | GO:0001533 | cornified envelope | 1.41E-70 | 43 |
| cellular component | GO:0005882 | intermediate filament | 4.65E-15 | 21 |
| cellular component | GO:0045095 | keratin filament | 5.55E-15 | 16 |
| cellular component | GO:0045111 | intermediate filament cytoskeleton | 1.28E-14 | 22 |
| molecular function | GO:0030280 | structural constituent of epidermis | 1.96E-12 | 8 |
| molecular function | GO:0004867 | serine-type endopeptidase inhibitor activity | 6.39E-09 | 11 |
| molecular function | GO:0004866 | endopeptidase inhibitor activity | 5.97E-08 | 13 |
| molecular function | GO:0030414 | peptidase inhibitor activity | 9.47E-08 | 13 |
| molecular function | GO:0061135 | endopeptidase regulator activity | 9.47E-08 | 13 |
| molecular function | GO:0061134 | peptidase regulator activity | 1.27E-07 | 14 |
| molecular function | GO:0005149 | interleukin-1 receptor binding | 3.89E-07 | 5 |
| molecular function | GO:0047498 | calcium-dependent phospholipase A2 activity | 5.61E-07 | 5 |
| molecular function | GO:0102567 | phospholipase A2 activity (consuming 1,2-dipalmitoylphosphatidylcholine) | 2.50E-06 | 5 |
| molecular function | GO:0102568 | phospholipase A2 activity consuming 1,2-dioleoylphosphatidylethanolamine) | 2.50E-06 | 5 |
| molecular function | GO:0050786 | RAGE receptor binding | 4.15E-06 | 4 |
| molecular function | GO:0004623 | phospholipase A2 activity | 3.53E-05 | 5 |
| molecular function | GO:0016755 | transferase activity, transferring amino-acyl groups | 3.63E-05 | 4 |
| molecular function | GO:0008236 | serine-type peptidase activity | 4.43E-05 | 12 |
| molecular function | GO:0017171 | serine hydrolase activity | 5.09E-05 | 12 |
| molecular function | GO:0004857 | enzyme inhibitor activity | 5.47E-05 | 14 |
| molecular function | GO:0016298 | lipase activity | 7.31E-05 | 8 |
| molecular function | GO:0004252 | serine-type endopeptidase activity | 9.15E-05 | 11 |
| molecular function | GO:0052689 | carboxylic ester hydrolase activity | 0.000138 | 8 |
| molecular function | GO:0070851 | growth factor receptor binding | 0.000591 | 7 |
| KEGG pathway | hsa00591 | Linoleic acid metabolism | 1.33E-07 | 6 |
| KEGG pathway | hsa00592 | alpha-Linolenic acid metabolism | 1.91E-06 | 5 |
| KEGG pathway | hsa00565 | Ether lipid metabolism | 2.67E-06 | 6 |
| KEGG pathway | hsa00590 | Arachidonic acid metabolism | 1.51E-05 | 6 |
| KEGG pathway | hsa05150 | Staphylococcus aureus infection | 2.36E-05 | 6 |
| KEGG pathway | hsa04657 | IL-17 signaling pathway | 0.001189 | 5 |
| KEGG pathway | hsa00564 | Glycerophospholipid metabolism | 0.001436 | 5 |
| KEGG pathway | hsa04060 | Cytokine-cytokine receptor interaction | 0.003581 | 8 |
| KEGG pathway | hsa05340 | Primary immunodeficiency | 0.003872 | 3 |
| KEGG pathway | hsa04975 | Fat digestion and absorption | 0.005184 | 3 |
| KEGG pathway | hsa04270 | Vascular smooth muscle contraction | 0.005471 | 5 |
